# Unveiling the metabolic potential of two soil-derived microbial consortia selected on wheat straw

**DOI:** 10.1038/srep13845

**Published:** 2015-09-07

**Authors:** Diego Javier Jiménez, Diego Chaves-Moreno, Jan Dirk van Elsas

**Affiliations:** 1 Department of Microbial Ecology, Groningen Institute for Evolutionary Life Sciences, University of Groningen. Nijenborgh 7, 9747AG. Groningen, The Netherlands; 2 Helmholtz–Centre for Infection Research, Inhoffenstr 7, 38124. Braunschweig, Germany

## Abstract

Based on the premise that plant biomass can be efficiently degraded by mixed microbial cultures and/or enzymes, we here applied a targeted metagenomics-based approach to explore the metabolic potential of two forest soil-derived lignocellulolytic microbial consortia, denoted RWS and TWS (bred on wheat straw). Using the metagenomes of three selected batches of two experimental systems, about 1.2 Gb of sequence was generated. Comparative analyses revealed an overrepresentation of predicted carbohydrate transporters (ABC, TonB and phosphotransferases), two-component sensing systems and β-glucosidases/galactosidases in the two consortia as compared to the forest soil inoculum. Additionally, “profiling” of carbohydrate-active enzymes showed significant enrichments of several genes encoding glycosyl hydrolases of families GH2, GH43, GH92 and GH95. Sequence analyses revealed these to be most strongly affiliated to genes present on the genomes of *Sphingobacterium*, *Bacteroides*, *Flavobacterium* and *Pedobacter spp*. Assembly of the RWS and TWS metagenomes generated 16,536 and 15,902 contigs of ≥10 Kb, respectively. Thirteen contigs, containing 39 glycosyl hydrolase genes, constitute novel (hemi)cellulose utilization loci with affiliation to sequences primarily found in the Bacteroidetes. Overall, this study provides deep insight in the plant polysaccharide degrading capabilities of microbial consortia bred from forest soil, highlighting their biotechnological potential.

The recent thrust to use plant waste such as straw for the production of compounds such as biofuel, plastics and intermediates for pharmaceuticals has raised major questions about the efficiency of lignocellulose deconstruction driven by microorganisms^1^,^2^. Moreover, the physical-chemical structures of different plant remains may vary considerably, hampering efforts to design strategies to completely degrade lignocellulose across plant waste sources^3^. One major source of straw is wheat, which contains, on average, 21% lignin, 28% hemicellulose and 36% cellulose^4^. Heat treatment of wheat tissue results in a softening of the lignin part, turning the (hemi)cellulose moiety more amenable to enzymatic digestion^5^. However, heating produces trace amounts of process-inhibiting furanic compounds, next to sugars, small polysaccharides, acids and ash^6^. Thus, in the light of the complexity of the (wheat) waste source, efficient bioconversion may depend on the synergistic action of diverse enzymes, such as hemicellulases, endo/exoglucanases and β-glucosidases. Hemicellulases include a variety of enzymes with different activities, such as mannosidases, arabinofuranosidases, fucosidases, galactosidases and xylanases. These glycosyl hydrolases (GH) act directly on the three major hemicellulose structures xylan, xyloglucan and galacto(gluco)mannan^7^. Hemicellulases, by nature, are known to enhance the conversion of lignocellulosic biomass to monosaccharides, thus improving the action of commercial cellulase-based enzyme cocktails^8^,^9^.

Unfortunately, we hitherto understand very little about the intricacies of lignocellulose biodegradation processes in microbial consortia. A key assumption about the mechanisms in such consortia is that the Darwinian principle of survival of the fittest reigns. Thus, sets of “locally fit” microorganisms in relative abundances that are appropriate for efficient biodegradation are predicted to be obtained at each time point in the process. A “matured” consortium then presumably contains all of the microbial members that have been, or are, important for the biodegradative process. Based on such assumptions, analyses of such matured consortia via metagenomics^10^,^11^ offer exciting possibilities to foster our understanding of the biodegradative process. In such analyses, the interplay of biodegradative next to other (supporting) processes may be unveiled, shedding light on the intricate ways in which the microbes that constitute the consortium interact with their environment and among themselves. In addition, genes that encode novel enzymes may become accessible, improving our knowledge on the biodegradative capacity of the individual members of the selected microbial communities^12^,^13^,^14^.

Current analyses of metagenomics-based datasets rely strongly on alignment of sequences against those present in databases. In the “Carbohydrate-Active Enzyme database” (CAZy), enzymes that hydrolyze diverse carbohydrates are classified into different GH families on the basis of their amino acid sequences^15^. Examination of the CAZy database reveals enormous sequence diversity, even within each gene family. On top of that, every new study on natural systems is expected to add more diversity to the database, as evidenced by recent work on the microbiomes of switchgrass-adapted compost^16^, herbivorous insects^17^,^18^, poplar wood chips^19^, buffalo rumen^20^ and snail guts^21^. To foster our understanding of the interplay of the different enzymatic processes in biodegradative microbial communities, it is important to reduce the diversity. Lignocellulose-based enrichments constitute an excellent vehicle to achieve this aim. Recently, Wongwilaiwalin *et al.*^22^ analyzed a rice straw-degrading microbial consortium enriched from sugarcane bagasse. Nine hundred and fifty seven GH-encoding genes were found, the most abundant ones belonging to the GH2, GH3 and GH43 families. Also, Deangelis *et al.*^23^ compared the metagenomes of two switchgrass-bred consortia (one of them amended with Fe). Enrichments of the GH65 family, sugar transporters (ABC systems), oxidoreductases and [Fe-S] binding proteins were found in the Fe-amended consortia. However, both studies did not address the levels of enrichment of such genes as compared to the microbial source nor did they analyze the genetic backgrounds of the identified GH-encoding genes. The latter analysis might allow the detection of metabolic pathways, regulatory mechanisms and substrate sensing/transport functions.

In previous work, we obtained two microbial consortia from forest soil by sequential transfers on *i*) untreated and *ii*) heat-treated wheat straw. Both consortia grew efficiently in each step and showed (hemi)cellulolytic enzymatic activities in the extracellular fractions^24^,^25^. In order to analyze the lignocellulolytic machineries present in the two consortia, we performed whole-metagenome analyses of selected consortia in a time course. We hypothesize that the microbial consortia analyzed were or became fine-tuned with respect to the utilization of resources from the wheat straw. The data from this study foster our understanding of the ecology of straw degradation and straw-based selection. They also give insight into the metabolic potential present in the resulting microbial consortia, opening up possibilities for further ecological and biotechnological studies.

## Results

### Selection of microbial consortia and production of metagenomic DNA

To foster our understanding of the genetic make-up of the “phylogenetically-stable” lignocellulose-degrading consortia, two biodegradative consortia, denoted RWS (bred on untreated wheat straw) and TWS (bred on heat-treated wheat straw), were selected, each at three time points. In addition, the source community (forest soil-derived) was selected as a comparator. Based on preliminary results from Fourier transformed infrared spectroscopical (FTIR) analyses, we obtained evidence for slightly higher percentages of lignin, hemicellulose and cellulose in the RWS (untreated wheat straw) compared with TWS (heat treated) substrate (data not shown).

Metagenomic DNA from all microbial consortia was successfully obtained at a rate of, on average, about 0.75 μg/ml culture (containing roughly 10^8^ bacterial cells/ml). A calculation of the extraction efficiency revealed that >90% of the bacterial cells in these had been captured into the extracts^26^. Illumina Mi-Seq sequencing of the metagenomes of the seven samples (forest soil-derived inoculum: FS1; transfer 1, 3 and 10 in RWS: 1W, 3W and 10W; and transfer 1, 3 and 10 in TWS: 1T, 3T and 10T) yielded a total of 5,067,095 sequence reads (average length 272 bp) that passed the quality criteria used, with a range of 385,175 to 1,026,339 reads per sample. The total genomic information (Mb) thus obtained for each metagenome was 136.82 (FS1), 170.58 (1W), 293.17 (3W), 112.23 (10W), 238.95 (1T), 221.46 (3T) and 198.10 (10T). On this basis, calculated coverage values for these respective metagenomes were 0.10, 0.39, 0.65, 0.28, 0.59, 1.23 and 1.05. The average G + C contents per metagenome showed strongly reduced values, of <50%, in the selected consortium samples as compared to that of the source community (60 ± 9%). Thus, G + C values measured in the latest enrichments were 41 ± 12% and 49 ± 13%, for 10W and 10T, respectively.

### Taxonomic assignment based on total protein-encoding and bacterial 16S rRNA gene sequences

MG-RAST-based^27^ annotation analyses of the reads in all metagenomes showed that around 70% of those in the RWS and TWS datasets had homologs on bacterial genomes (RefSeq database). The remaining reads (roughly 30%) were similar to unannotated genomic regions (e.g. unknown DNA or intragenic sequences). The total reads that could be affiliated to Eukarya with the existing tools was consistently low (<0.001% across all metagenomes). In contrast to the above, only 38% of the reads from the FS1 source community was predicted to encode proteins. On the basis of an analysis of the affiliation of the collective protein-encoding sequences, the bacterial community structures (at genus level) turned out to be quite different (r^2^ = 0.480) between the RWS and the TWS consortia (Supplementary Fig. S1). They were also different from that of the FS1 source community. Remarkably, in the FS1 metagenome, one 16S rRNA sequence was retrieved per 11,276 reads, whereas in the RWS as well as TWS consortium ones, the ratios were 1/800, 1/820, 1/618, 1/673, 1/576 and 1/628 for 1W, 3W, 10W, 1T, 3T and 10T, respectively. Considering the phylogenetic affiliations, some differences were observed between the taxonomic profile based on protein-encoding and the bacterial 16S rRNA gene sequences. For example after transfer-3, a predominance of *Sphingobacterium* sequences (35% versus 3% of *Klebsiella*) was found in the 16S rRNA-based classification, while *Klebsiella*-like sequences were most abundant in the protein-encoding gene approach (~15% versus 10% of *Sphingobacterium*) (Supplementary Fig. S1).

### Functional profiles — evidence for a dominance of ABC transporters and TonB-dependent receptors

**Figure 1:**
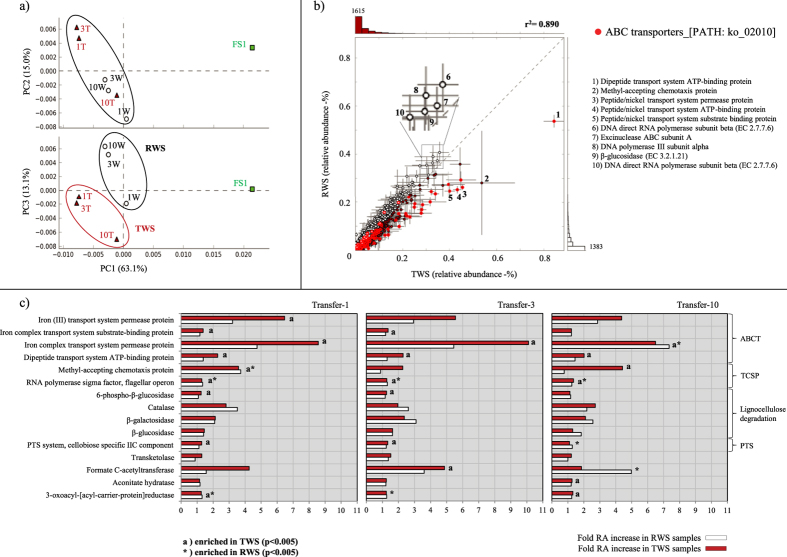
Functional profile of the metagenomes based on KEGG identifiers. (**a**) PCA of the seven metagenomes using the lower hierarchical functional level; (**b**) Relative abundance (%) of microbial functions and pairwise comparison between RWS (1W, 3W and 10W) and TWS (1T, 3T and 10T) samples, red dots represent functions within the ABC transporters; (**c**) Highly enriched specific functions (compared with soil inoculum FS1) along three sequential transfers (fold-increase). Abbreviations: ABC transporters (ABCT), two-component system proteins (TCSP) and phosphotransferase systems (PTS).

In order to obtain a robust metabolic profile, two databases were used for the analyses (KEGG and SEED). Based on principal components analyses (PCA) of the KEGG-based functional profiles, the TWS metagenome samples 1T, 3T and 10T all clustered together, suggesting stabilization and preservation of function as from transfer-1. Quite remarkably, these metagenomes still revealed considerable levels of similarity with those from RWS (r^2^ = 0.890). Thus, both consortia showed largely similar functional profiles at three time points, which were clearly different from those of the FS1 community (Fig. 1a,b). Pairwise analyses revealed that FS1 and RWS correlated at r^2^ = 0.608 and FS1 and TWS at r^2^ = 0.513.

Close examination of the differences between the RWS and TWS consortium metagenomes on the one hand and the FS1 community one on the other hand showed several significantly enriched functions (p < 0.005), at KEGG orthology level 2. These were membrane transport systems (mainly ABC transporters) next to systems for the metabolism of cofactors and vitamins, cell motility and glycan biosynthesis (Supplementary Fig. S2). ABC transporters (ABCT) (9.5 ± 0.9% and 13 ± 0.7% relative abundance -RA- in RWS and TWS samples, respectively, versus ~8.5% in FS1) and two-component system proteins (TCSP) (6.0 ± 0.25% RA in both consortia, versus ~5% in FS1) were the most abundant functions in both consortia. The most overrepresented (p < 0.005) specific functions, after transfer-3, in the RWS and TWS metagenomes were iron transport system permease proteins (up to 10-fold RA increases), formate C-acetyltransferases (~2–5-fold RA increase). In addition, methyl-accepting chemotaxis proteins and phosphotransferase system (PTS)-cellobiose-specific IIC components were also enriched. Interestingly and with respect to the biodegradative process, sequences predicted to encode enzymes involved in lignocellulose degradation were indeed also overrepresented in the RWS and TWS consortia as compared to the FS1 community. This included (partial) genes encoding β-glucosidases (1.62, 1.83, 1.62 and 1.29 -fold RA increases in 3W,10W, 3T and 10T, respectively), β-galactosidases (3.09, 2.57, 2.36 and 2.09 -fold RA increases in 3W, 10W, 3T and 10T, respectively) and catalases (2.61, 2.18, 1.96 and 2.72 -fold RA increases in 3W, 10W, 3T and 10T, respectively) (Fig. 1c).

**Figure 2:**
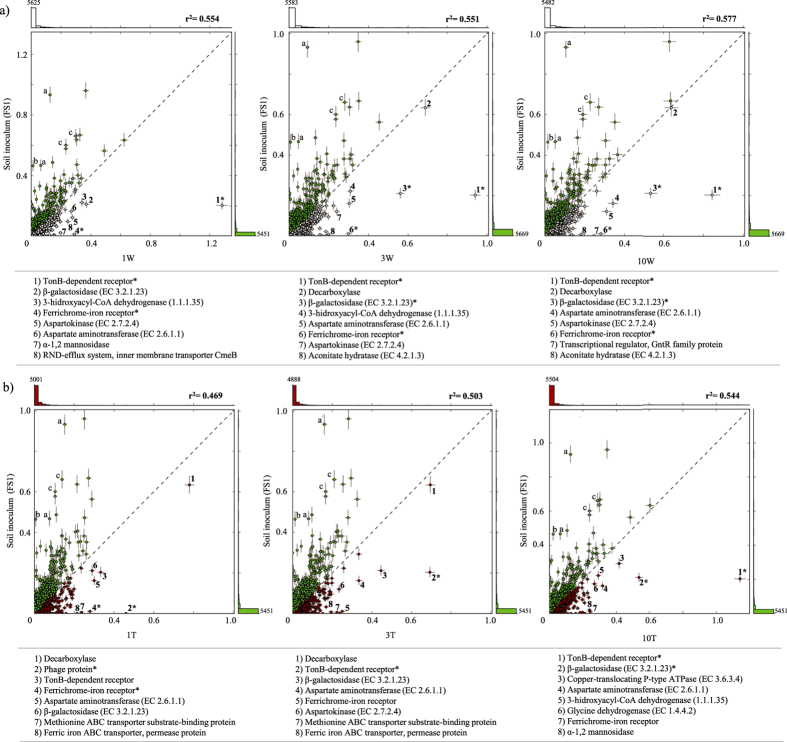
Comparison of functional profiles (percentage of relative abundance of SEED identifiers at lower hierarchical level) between the soil inoculum FS1 and metagenomes of the three sequential transfers of (**a**) RWS and (**b)** TWS. Numbers: are the selection of the eight most overrepresented functions in the consortial metagenomes. Asterisks (*) represent functions that were most enriched in RWS and TWS samples (p < 0.005). Letters a (adenylate cyclase), b (carbon monoxide dehydrogenase) and c (and cobalt/cadmium/zinc resistance proteins) correspond to the most deselected functions (p < 0.005).

Comparative SEED-based analyses of the RWS and TWS consortium metagenomes versus that of the FS1 community showed that the most enriched specific genes (p < 0.005) in the 3W, 10W and 3T metagenomes were those for utilization of monosaccharides (2.06 ± 0.16 -fold RA increase) as well as iron acquisition (4.53 ± 0.26 -fold RA increase). In the 10T one, genes for proteins involved in iron acquisition were also strongly enriched (5-fold RA increase) (Supplementary Fig. S3). In the 3W and 10W metagenomes, genes for TonB-dependent receptors (TBR) (4.63 and 4.15 -fold RA increases, respectively), and, to a lesser extent, for ferrichrome-iron receptors and β-galactosidases were enriched in comparison to the FS1 one (p < 0.005). In contrast, genes for decarboxylases were highly abundant in FS1, 3W and 10W (~0.65% RA) (Fig. 2a). For TWS, TBR (3.4 and 5.6 -fold increases in 3T and 10T, respectively), ferrichrome-iron receptors and β-galactosidases were enriched in the 3T metagenome, whereas decarboxylases were depleted at 10T (Fig. 2b). On the other hand, three specific functions, i.e. adenylate cyclase, carbon monoxide dehydrogenase and cobalt/cadmium/zinc resistance proteins, were strongly deselected (p < 0.005) in the two systems.

### Genes predicted to be involved in lignocellulose degradation

**Figure 3:**
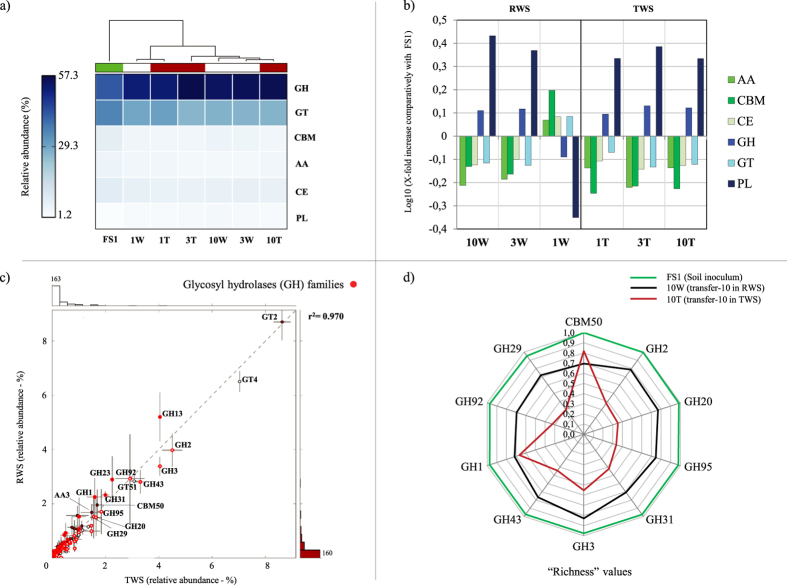
Carbohydrate-active functional profiles. (**a**) Heat map of the relative abundance (%) of each CAZy class (AA, CBM, CE, GH, GT and PL) in each metagenome; (**b**) Log_10_ X-fold increase of each CAZy class in RWS and TWS samples comparatively to the soil inoculum (FS1); (**c**) Pairwise comparison between RWS (1W, 3W and 10W) and TWS (1T, 3T and 10T) samples, red dots: are functions belonging to glycosyl hydrolases (GH) families; (**d**) “Richness” values (number of clusters at 97% nucleotide identity over the total retrieved reads in each family) of the most enriched CAZy families at transfer-10 (10W and 10T) and comparison with the soil inoculum (FS1).

In order to detect genes, or parts thereof, involved in lignocellulose deconstruction, we performed BLASTX of all metagenome reads against the CAZy database. The results show that, after transfer-3, the carbohydrate-active genetic profiles were stable and consistent in the RWS and TWS metagenomes (Fig. 3a). The most enriched functions at the last transfers (compared with FS1) were polysaccharide lyases (PL) (0.43 and 0.33 log X-fold in 10W and 10T, respectively) and GH (0.11 and 0.12 log X-fold in 10W and 10T, respectively) (Fig. 3b). The correlation (r^2^) between the RWS and TWS consortium metagenomes was 0.970. Specific enzyme families that abounded in the TWS and RWS metagenomes were glycosyl transferase (GT) families 2 (~8.5% RA) and 4 (~6.5% RA), followed by GH2, GH13 and GH3 (Fig. 3c; Table 1).

An overall examination of the data revealed the presence of a high diversity of predicted enzymes, with respect to the number and diversity of CAZy families. To reduce the complexity of the data, we selected a “top-60” of the most enriched CAZy families in both consortia for further analyses. Data on count matches, RA and log_10_ fold increases for each consortium and transfer are presented in the Supplementary Table S1. As compared to the FS1 source community, some genes of particular families were highly enriched (≥1 log_10_ unit) in both derived consortia, albeit at rather low (≤0.3) RA levels. Specifically, these were genes for the GH117, GH50, GT8 and PL17 family proteins. The most abundant (approximately >1.5% RA) enriched (p <0.005) families in the two consortia were GH1, GH2, GH3, GH20, GH29, GH31, GH43, GH92, GH95 and CBM50. Remarkably, enrichment of these was already apparent as from transfer-1 (Supplementary Fig. S4), suggesting the occurrence of a strong selective effect early on in the consortium enrichments. Specifically, reads falling in families GH1, GH2, GH31 and CBM50 were enriched along the transfers in the RWS and those of families GH2, GH20, GH29, GH43, GH92 and GH95 in the TWS metagenomes. In contrast, reads for families PL17, GH50, GH3 and GH13 were deselected or showed a neutral behavior along the transfers (Table 1).

### CAZy families enriched at transfer-10 - tracking richness and microbial source

**Figure 4:**
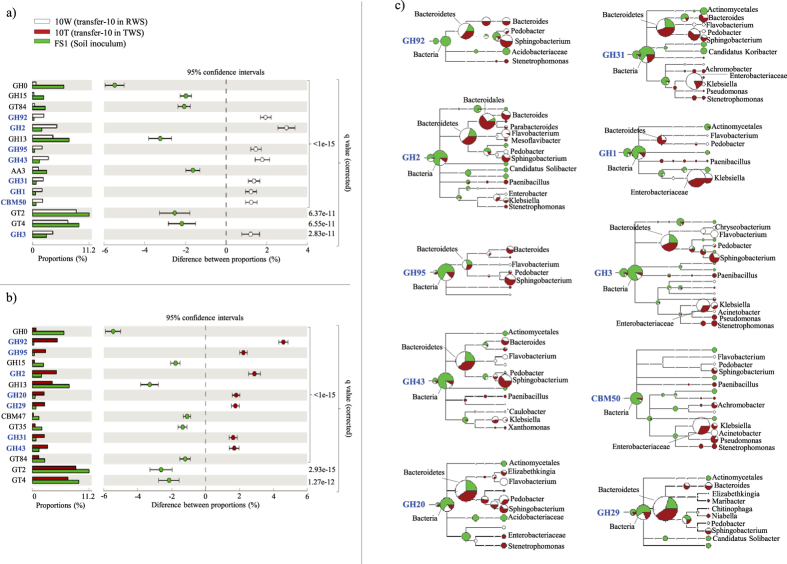
Differentially enriched CAZy families (p < 0.005, 95% confidence intervals) between FS1 and (a) 10W and (b) 10T metagenomes; (c) Taxonomi**c** affiliation of reads belonging to families GH92, GH2, GH95, GH43, GH20, GH31, GH1, GH3, GH29 and CBM50, in 10W, 10T and FS1, using the Lowest Common Ancestor (LCA) algorithm.

The reads of overrepresented CAZy families at transfer-10 (stable community) (Fig. 4a,b) were clustered at 97% nucleotide identity cut-off and taxonomically classified. Using the normalized numbers of clusters in each family, we observed that reads assigned to all enriched CAZy families (except CBM50) in 10T showed lower “richness” values than those in 10W and FS1. Richness was defined as the number of clusters at 97% nucleotide identity over total retrieved reads in each family. Families GH1, GH31, CBM50 and GH92 showed “richness” values between 0.71 to 0.69 in 10W, but GH20, GH95, GH92 and GH29 had “richness” values between 0.35 to 0.29 in 10T (Fig. 3d). Based on the taxonomic classification by the lowest common ancestor (LCA) algorithm^28^ (Fig. 4c), the GH2, GH20, GH29, GH43, GH92, GH95 reads were mainly assigned to genomes of members of the Bacteroidetes (e.g. *Sphingobacterium*, *Bacteroides*, *Flavobacterium* and *Pedobacter*). Moreover, sequences that belonged to the GH1, GH3, GH31 and CBM50 families were mostly affiliated with organisms from within the Enterobacteriaceae, especially within *Klebsiella*. The taxonomic affiliation of the GH retrieved from transfer-1 and -3, in both consortia, showed similar results, indicating that the enrichments selected for GH assigned to members of the Bacteroidetes and Enterobacteriaceae.

Additionally, in order to explore whether the reads make part of any specific genetic region, we performed an assembly (contigs > 750 bp as a cut-off) of all reads in each overrepresented family. This approach yielded no contigs for the FS1 metagenome, indicating great heterogeneity. From the 10W metagenome, 17, 12, 9, 8 and 5 contigs were produced for the GH2, GH92, GH43, GH31 and GH29 families, respectively. Between 28.03 to 59.38% of the total retrieved reads, in each of these last families, were used. In contrast, in the 10T metagenome, 88, 86, 82, 81 and 79% of the total retrieved reads in GH29, GH92, GH31, GH2 and GH43 were used to construct 12, 15, 7, 13 and 15 contigs, respectively (Supplementary Table S2). Contigs (or complete genes) with the highest read coverage were analyzed by BLASTX against the nr-NCBI database. All GH-encoding genes in 10T were thus identified to derive from members of the Bacteroidetes, especially *Sphingobacterium* spp. However, the most abundant GH1, GH3 and GH31-encoding genes in 10W were affiliated with predicted proteins of *Kluyvera ascorbata* (Table 2).

### Contigs containing gene clusters predicted to be involved in (hemi)cellulose degradation

**Figure 5:**
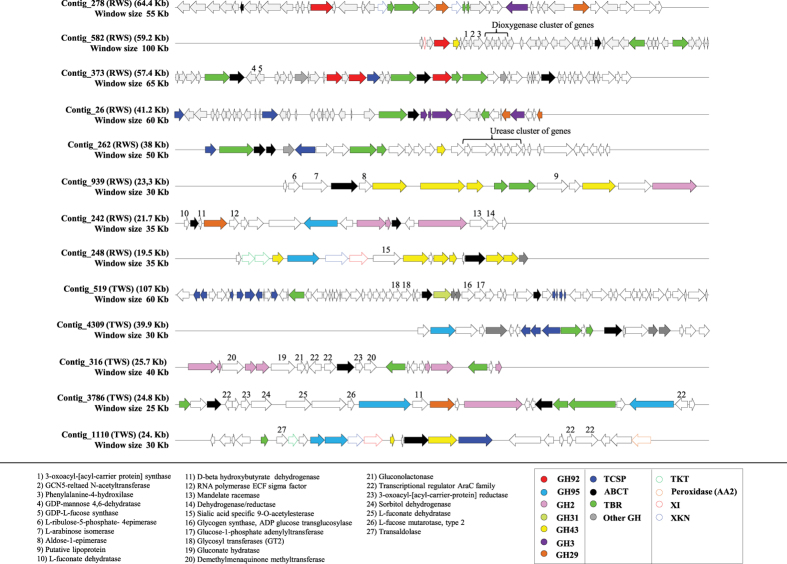
Graphical representation of thirteen novel Bacteroidetes HULs (hemicellulose utilization loci) recovered from the metagenome assemblages. Numbers represents annotated proteins that are flanked by glycosyl hydrolase (GH) genes. Abbreviations: Two-component system proteins (TCSP), ABC transporters (ABCT), TonB-dependent receptors (TBR), transketolases (TKT), xylose isomerase (XI) and xylulose kinase (XKN) genes.

Totals of 16,536 (contig mean length 3.6 Kb) and 15,902 (contig mean length 3.4 Kb) contigs were generated for the metagenomes obtained for the RWS and TWS consortia, respectively. Of these, 10,571 and 9,987 contigs, respectively for RWS and TWS, were >1,000 bp. Moreover, in RWS, 94 GH genes (specifically belonging to families GH2, GH3, GH29, GH31, GH43, GH92 and GH95) were detected in 70 contigs (≥5 Kb). In TWS, 75 GH were identified in 58 contigs (≥5 Kb) (Supplementary Table S3). Thirteen large contigs (≥10 Kb; G + C content between 35 to 42%; read coverage 14X to 30X) contained at least two GH genes. Based on CAZy annotations, in these contigs we identified a total of 39 GH genes. Interestingly, 11 of these showed an identity less than 60% to already described proteins (Table 3). Regarding the RAST annotations, 25, 19 and 20 genes encoding TBR, ABCT and TCSP, respectively, were detected in the selected 13 contigs (Fig. 5). The tetranucleotide frequencies (TTNF) and average nucleotide identities (ANIb) results showed that these contigs were mainly affiliated with regions on genomes of *Sphingobacterium*, *Bacteroides*, *Parabacteroides*, *Flavobacterium* and *Pedobacter*. However, the 13 contigs showed an ANIb < 72% and a correlation of TTNF less than 0.79, suggesting that these genomic fragments make part of as yet unknown (yet related) genomes (Supplementary Table S4). Seven contigs were presumed to belong to organisms affiliated with *Sphingobacterium* (contigs 278, 373, 26, 4309 and 1110), *Pedobacter* (contig 248) and *Parabacteroides* (contig 316), on the basis of BLAST analyses of the GH genes (Table 3). All contigs were found to contain at least one complete predicted operon. Contigs 242, 248, 1110, 4309 and 3786 contained operons/genes with predicted roles in xylan, xyloglucan and galacto(gluco)mannan degradation (Fig. 5). In addition, contigs 278 and 26 contained operons/genes involved in the bioconversion of cellobiose (GH3). Contig 939 contained genes encoding L-ribulose-5-phosphate 4-epimerase, L-arabinose isomerase, aldose-1-epimerase and a putative lipoprotein. Interestingly, in contigs 278, 582, 248 and 1110 we found genes involved in sugar metabolism steps, such as transketolases (TKT) (3), xylose isomerase (XI) (3) and xylulose kinase (XKN) (4). In contig 1110, one putative gene involved in lignin metabolism (cytochrome peroxidase - EC1.11.1.5) was found. Finally, six genes encoding transcriptional regulators of the AraC family were found in contigs 316, 3786 and 1110 (Fig. 5).

## Discussion

In this study, we analyzed 576 (RWS) and 658 (TWS) Mb of sequence information from two lignocellulose-bred microbial consortia, in comparison to a source community from forest soil (FS1). This allowed a robust comparative metagenomic analysis to be performed. To avoid biases, we used two different methodologies. First, unassembled sequences were used for taxonomic and functional assignments (using RefSeq, KEGG, SEED and CAZy), on the premise that such analyses would not “distort” the abundance and representation of the sequences of abundant species in the dataset^29^. In addition, we performed an assembly of reads in our metagenomes, allowing to recover complete genes and gene clusters or operons, and to study the arrangement of genes for the most overrepresented GH (compared with FS1). An estimation of the predicted abundance of the organisms carrying these gene clusters resulted in the contention that such organisms occurred among the top-5 members of these consortia.

Comparative analyses of the consortium metagenome datasets versus those from the source community first revealed that organisms with genomes with relatively low G + C content had been strongly selected in the consortia. This indicated a selection of fast-growing decomposer organisms with, on average, lower G + C% genomes, e.g. Proteobacteria and Bacteroidetes, and concurrent deselection of the high G + C positive members of the soil microbiota, e.g. Actinobacteria. The fact that a majority (around 70%) of the predicted protein-encoding genes, or parts thereof, in both consortia had homologs (>50% homology) on published bacterial genomes supported the contention of selection of bacterially-dominated degrading consortia. The differences between the percentages of mappable reads between the bred microbial consortia (~70%) and the soil inoculum (~38%) could be explained by the fact that soil contains high microbial diversity and DNA novelty compared with most enrichment cultures. The percentage of annotated reads in FS1 was similar to that found in previous soil metagenomics-based studies^30^,^31^. Remarkably, we found differences and fluctuations between the taxonomic profile based on protein-encoding and the bacterial 16S rRNA gene sequences in the metagenomes under study. However, it is known that major biases exist in both gene enumerations, as both 16S rRNA and functional gene copy numbers may vary, differently, across genomes. In addition, their accuracy also depends on the representation of the different taxonomic groups in a database^32^. Based on an average copy number of four 16S rRNA gene sequences per bacterial genome of 4 Mb, and a mean length of 1.5 Kb per gene copy, indeed roughly 1/1000 sequences are expected to represent 16S rRNA genes^33^,^34^. Hence, the ratio’s (1/618 to 1/820) obtained in the RWS and TWS consortia are indicative of largely bacterially-driven communities, whereas that in the FS1 source community (1/11,276) may have come about as a result of (1) undetectable “novel” 16S rRNA gene sequences, (2) the co-occurrence of a larger proportion of fungi, (3) a generally larger bacterial genome per cell (6–12 Mb) and (4) a generally lower number of rRNA gene copies per genome.

With respect to the functional profiles, ABCT and phosphotransferase systems function in the cellular uptake of sugars (e.g. cellobiose, cellodextrin, glucose, galactose and mannose). In addition, some ABCT can import key ions such as Sn, Fe and Co^35^,^36^. Interestingly, ABCT may be differentially expressed in *Clostridium*, *Thermobifida* and *Streptomyces* species in response to different lignocellulosic substrates^37^,^38^,^39^, indicating their involvement in transport of intermediate products of biodegradation or sugars. Deangelis *et al.*^40^ suggested that ABCT mediate - in *Enterobacter lignolyticus* - the transport of xylose and lignin-derived aromatic compounds into the cells. Moreover, ABCT were also abundantly present in a metagenome of the gut of a wood-feeding beetle (*Anoplophora glabripennis*)^41^. Overrepresentation of genes for these type of proteins was also evident in a tropical soil-microbial consortia selected by switchgrass^23^.

Regarding TonB-dependent receptors (TBR) (outer membrane proteins that are known for the active transport of Fe-siderophore complexes, vitamin B12 and plant carbohydrates in Gram-negative bacteria), in *Caulobacter crescentus*, expression is induced by xylose^42^. Blanvillain *et al.*^43^ reported that, in phytopathogenic and aquatic bacteria, TBR are involved in scavenging of plant sugars. Fernández-Gómez *et al.*^44^ showed that genes encoding several polysaccharide-degrading proteins (e.g. β-galactosidases or β-xylosidases) are located in close proximity to genes for TBR and transducers (especially in Bacteroidetes), suggesting an integrated regulation of adhesion to, and degradation of, polysaccharides that are attacked by the products of the neighboring genes. An interesting study, in *Gramella forsetii*, revealed that TBR also could work as anticipatory polysaccharide sensors^45^. With respect to the two-component system proteins (TCSP), these may sense carbohydrates using the sensing domain in the periplasm^46^. In *Clostridium cellulolyticum*, TCSP perceive the presence of extracellular soluble sugars and regulate most GH and associated ABCT^37^. In *Bacteroides*, these TCSP act similarly and regulate (hemi)cellulose utilization loci (HULs)^47^,^48^,^49^.

The overrepresentation of genes for ABCT, TBR and proteins involved in lignocellulose degradation (e.g. β-xylosidases) in RWS and TWS is consistent with the hypothesis that enhanced capacities are required in these consortia in order to facilitate the utilization of complex plant materials (e.g. hemicellulose) and transport the products (e.g. xylose) of various structures into the cells. Bacterial consortia were thus selected that metabolize the partially degraded plant material. In addition, the genomic machineries in these consortia (especially TCSP or TBR) apparently sense the activation of GH and ABCT in response to a wide range of environments, stressors and growth conditions, such the use of wheat straw as carbon source.

The functional assignments, in conjunction with the taxonomic profiles based on genes encoding proteins, as well as previous 16S rRNA gene and ITS pyrosequencing analysis^25^, indicate that the RWS and TWS consortia had distinct microbial community structures, but highly similar functional profiles. Clearly, function was a much stronger driver of community structuring than phylogeny. Similar results have been reported in the past for other microbial communities^50^,^51^. We observed that different functional profiles had emerged in comparison to the FS1 community, although functional overlaps still existed. However, the high percentage of sequences with no homology (in FS1) could mask the conservation of function between the consortia and the soil inoculum. It is likely that selective force, in relation to the complexity of the wheat straw, offered many microbial niches, giving origin to a still rather complex consortium in both enrichments.

**Figure 6:**
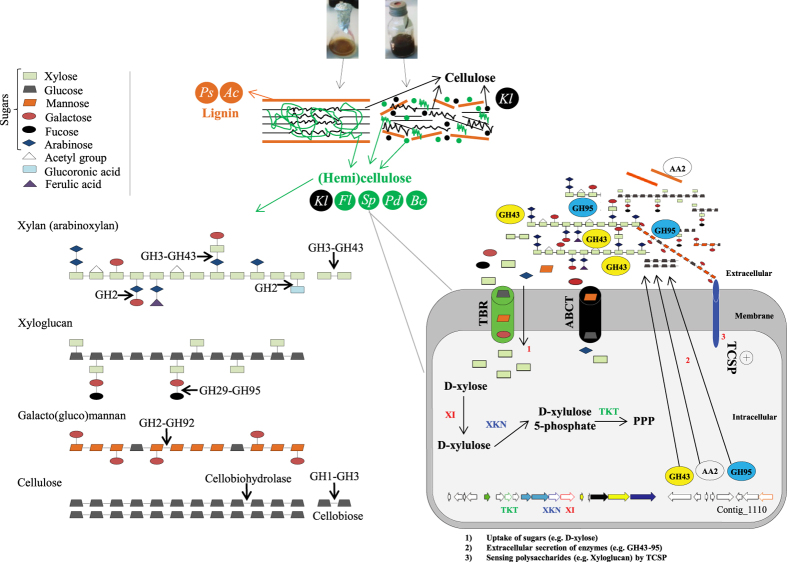
Graphical explanation of the presumed catalytic mode of action, on the major component of (hemi)cellulose, by the most enriched GH in our consortia (left). GH2 hydrolizes β-glycosidic bonds between galactose and its organic functional group; GH95 and GH29 are enzymes that hydrolyze Fuc-alpha1-2Gal linkages attached to the non-reducing ends of oligosaccharides; GH43 can act directly on xylan and release D-xylose and L-arabinose as main products. At the top: two different lignocellulose structures and microorganisms involved in their deconstruction (flask pictures were taken by the authors). Abbreviations: *Acinetobacter* (Ac), *Klebsiella*/*Kluyvera* (Kl), *Flavobacterium* (Fl), *Pseudomonas* (Ps) and *Sphingobacterium* (Sp). Right, partial metabolic reconstruction based on a theoretical (hemi)cellulose degradation pathway and uptake of sugars (intra - and extracellularly), in Bacteroidetes, using the contig_1110 genetic information. Abbreviations: Two-component system proteins (TCSP), ABC transporters (ABCT), TonB-dependent receptors (TBR), transketolases (TKT), xylose isomerase (XI) and xylulose kinase (XKN) and pentose phosphate pathway (PPP). Plus sign represent the regulation of the GH by the TCSP.

The CAZy profile data were consistent with those previously reported based on the function predictor PICRUSt^25^. This suggests that, for relatively simple communities, such predictions are consistent with “reality”, as also suggested recently^52^. In terms of enriched CAZy families, GH50 and GH117 constitute enzymes involved in the degradation of complex polysaccharides, such as agarose and neoagarobiose^53^,^54^. The role of these two GH families in lignocellulose deconstruction is still unknown, however their enrichment points to a role in plant biomass degradation. A subset of the enriched enzymes in our consortia is capable of degrading different structures in the lignocellulose materials. For example, GH1, GH2 and GH3 (mostly β-glucosidases and β-galactosidases) are mainly involved in cellobiose and oligosaccharide deconstruction. The α-mannosidases (GH92), α-fucosidases (GH95 and GH29), α-arabinofuranosidases and β-xylosidase (GH43) are the most important hemicellulose active exo-enzymes that catalyze the hydrolysis of plant polysaccharides in agricultural waste^7^ (Fig. 6). High levels of these families were reported in a microbial community decomposing poplar wood chips, especially sourced from Bacteroidetes members^19^. GH92 family genes have been shown to be abundantly present in a buffalo rumen metagenome^55^. Interestingly, previous enzymatic analysis showed that the β-glucosidases, β-galactosidases and β-xylosidase were highly active in the secretome of both our consortia at transfer-10^25^, confirming the presence of these types of genes and their expression.

On the other hand, based on “richness” values and the contigs constructed from the reads that came from enriched GH families in transfer-10, we hypothesize that strong selection, yielding “less niches or less complex and specific carbon sources”, occurred for the 10T and 10W systems, compared with FS1. In addition, a reduction of the functional “richness” values was more evident in 10T than in 10W, confirming that pretreatment reduces the complexity of the substrate and can introduce a stronger, or narrower, selection, which is possibly also based on the presence of furanic compounds^24^. The low “richness” values and the classification, at “high” taxonomy ranking (e.g. family GH29 in 10T), might constitute evidence for selection of specific novel proteins. Finally, the large enrichment of GH families associated with Bacteroidetes (i.e. *Sphingobacterium*, *Bacteroides*, *Flavobacterium* and *Pedobacter*) types at transfer-10 indicated these taxa as prime agents in the deconstruction of the hemicellulose part on the wheat straw. This stood in contrast to the presumed role of Enterobacteriaceae (i.e. *Klebsiella* and/or *Kluyvera* types) which may act mainly on the cellulose structures, and possibly also the lignin^40^ (Fig. 6). We hypothesize that soils with a majority of Bacteroidetes have elevated levels of metabolic capabilities to degrade plant polysaccharides, especially hemicellulose.

In lignocellulolytic microbial consortia of reduced complexity, genome pieces and/or gene clusters might be assembled at the coverage levels used by us, allowing the description/detection of partial metabolic pathways related with plant waste deconstruction. Our metagenome assembly yielded thirteen putative novel Bacteroidetes HULs. This apparent “bias” towards Bacteroidetes is consistent with the reported relative dominance of this taxon in both our selected microbial consortia^25^. The collective contigs contained genes for 39 GH involved in (hemi)cellulose degradation, of which 11 might encode novel proteins based on the low identity percentage with database entries. Minor differences in the numbers of detected GH were observed between the CAZy and RAST based annotations (e.g. contigs ID 939, 248 and 316), which might be explained by differences in the two gene-calling systems used.

Interestingly, the GH detected in the 13 retrieved HULs were mostly flanked by TBR, TCSP, ABCT and genes involved in sugar metabolism (e.g. TKT, XKN and XI). Recently, four types of regulatory systems were proposed to be involved in transcriptional regulation of genes for polysaccharide degradation in *Bacteroides* sp.: *i*) Sus-like regulators (or TBR), *ii*) TCSP, *iii*) extra cytoplasmic function (ECF) sigma/anti-sigma factors and *iv*) AraC family regulators^46^,^56^. In *B. thetaiotaomicron*, SusC is a member of the TBR family specialized in the transport of oligosaccharides from the outer membrane into the periplasmic space^44^. Moreover, the presence of genes involved in sugar metabolism, in our retrieved contigs, was indicative of partial pathways encoded by these genomic fragments. Adav *et al.*^38^ revealed an up-regulation of XI in *Thermobifida fusca* when grown on different lignocellulosic biomass. Interestingly, the heterologous expression of L-ribulose-5-phosphate 4-epimerase, L-arabinose isomerase, aldose-1-epimerase (found in contig 939) could improve sugar utilization and production of ethanol in *Zymomonas* and *Saccharomyces* species^57^,^58^. Based on the cluster of genes in contig 1110, the theoretical and hypothetical pathway could be: D-xylose produced from the hydrolysis of xyloglucan by GH43, recognized by TBR or TCSP and transported into cells by ABCT. Once inside the cell, XI converts it to D-xylulose and subsequently to D-xylulose 5-phosphate by XKN. D-xylulose 5-phosphate then enters the pentose phosphate pathway with the help of the TKT (Fig. 6).

Thus, our data demonstrate the presence of partial pathways related with plant biomass deconstruction and sugar (e.g. xylose, glucose, arabinose and fucose) transport and metabolism. As a prospect, abundant and enriched enzymes, such as mannosidases (GH92), arabinofuranosidases, xylanases (GH43), fucosidases (GH95 and GH29) and galactosidases (GH2) can be extracted from the HULs, custom-synthesized, codon-optimized and expressed in a suitable host. This will generate novel efficient enzyme cocktails that can enhance the activity of currently-used cellulolytic enzymes^8^,^9^. Overall, the data from this study allow us to clarify the consortial metabolic capacities, including the unraveling of lignocellulose-active enzymatic machineries. This demonstrates the biotechnological potential of the two soil microbiome derived consortia selected by wheat straw.

## Materials and Methods

### Lignocellulolytic microbial consortia and metagenome sequencing

Breeding of the microbial consortia has been reported before^24^. Briefly, cell suspensions were prepared by adding 10 g of sampled soil to 250 ml flasks containing 10 g of sterile gravel in 90 ml of mineral salts medium (MSM). The flasks were shaken for 20 min at 250 rpm. The cell extracts (250 μl) were then introduced into triplicate flasks containing 25 ml of MSM supplemented with 1% of *i*) “raw” wheat straw (RWS) and *ii*) heat-treated (240 °C, 1 h) wheat straw (TWS). Subsequently, flasks were incubated at 25 °C with shaking (100 rpm). Once the systems reached 7 to 8 log cells/ml (between 6 and 8 days), aliquots (25 μl) were transferred to 25 ml of fresh MSM containing the same substrates. Samples from the soil suspension (FS1) and from the triplicate RWS and TWS flasks were taken after 1, 3 and 10 transfers (n = 21). The selection of the samples was based on previous bacterial PCR-DGGE results^24^. Metagenomic DNA was extracted from the FS1 (n = 3) and from the selected RWS and TWS samples (n = 18) using the UltraClean Microbial DNA Isolation Kit (MoBio® Laboratories Inc., Carlsbad, CA, USA), according to the manufacturer’s instructions. Based on previous bacterial 16S rRNA gene and fungal ITS1 diversity analyses (DGGE and pyrosequencing), the latter samples showed consistent microbial structure and composition per time point^24^,^25^. The triplicate DNAs from the FS1 and from each selected RWS and TWS consortia were pooled, yielding a total of 7 metagenomes. The DNA samples were then subjected to Illumina Mi-Seq v2 sequencing (250 bp paired-end reads) at LGC Genomics (Berlin, Germany).

### Sequence processing and annotation of unassembled sequences

A total of 5,298,311 unassembled sequences (reads) were uploaded to the MG-RAST v3.1.2 server^27^. Overlapping sequence pairs were matched, and non-overlapping reads retained as individual reads, after which dereplication was performed. Duplicate read based inferred sequencing error estimation and quality trimming (phred score <20) were done using default settings in MG-RAST. The coverage values of each metagenome was calculated based on the number of bacterial 16S rRNA OTUs at 97% cut-off value (338, 108, 112, 100, 102, 45 and 46 for FS1, 1W, 3W, 10W, 1T, 3T and 10T, respectively)^25^, using as a reference 4 Mb per bacterial genome. Gene predictions were done using the FragGeneScan software and subsequently the predicted proteins were annotated based on BLASTX searches against the RefSeq, KEGG and SEED databases using an e-value cutoff of 1e-15, a minimum alignment length of 50 amino acids and a minimum identity of 50%. In order to retrieve 16S rRNA gene sequences from the metagenomes, we performed a BLASTN search against the RDP database using an e-value cutoff 1e-15, a minimum alignment of 200 base pairs (bp) and a minimum nucleotide identity of 90%. The 16S rRNA gene sequences were taxonomically classified using the Classifier software with a confidence threshold of 50%^59^,^60^. The total metagenome sequences are publically accessible under the MG-RAST project code 7220 (Metagenome IDs 4547279.3 to 4547285.3).

### Detection and compositional profile of genes involved in plant waste deconstruction

Genes, or parts thereof, involved in lignocellulose degradation were detected using as a starting point the reads (quality filtered and trimmed) obtained by MG-RAST. Annotation was performed via BLASTX against the CAZy database v3 (downloaded from dbCAN site)^61^,^62^ using an e-value cutoff 1e-15. Filtering was done based on a measure that relates the coverage and the identity percentage (ratio) (alignment length × % identity/query length) of >30%. To evaluate the relative abundance (RA) of reads per family, the counts were normalized using the total numbers of quality reads (matched in the database) per metagenome. Moreover, the RA of reads of each class (AA, CBM, CE, GH, GT and PL) and family was compared between the metagenomes from the selected consortia (1W, 3W, 10W, 1T, 3T and 10T) as well as the FS1. Fold changes were calculated based on the normalized RA values (fold increase = RA consortia/RA of FS1) and then Log_10_ transformed.

Unassembled sequences belonging to CAZy families CBM50, GH1, GH2, GH3, GH20, GH29, GH31, GH43, GH92 and GH95 were extracted from FS1, 10W and 10T (stable consortia), re-annotated (in-house) using BLASTX searches against the nr-NCBI database and clustered at 97% of nucleotide identity using the CD-HIT software^63^. The BLAST results were loaded into MEGAN v5 software and classified taxonomically according to suggested parameters for the Lowest Common Ancestor algorithm (LCA) (maximum number of matches per read: 10; min support: 5; min score: 35; max expected 0.01; min complexity 0.3; and top percent: 10)^28^. To calculate the RA and the relative differences of read “richness” value, the counts and clusters were normalized using the total numbers of reads within each selected family. In addition, reads extracted in each family were assembled using the CLC Genomics Workbench v4.0.3 software (CLC Bio, Cambridge, MA, USA).

### Comparisons and statistical analysis of unassembled metagenome data

Data from MG-RAST and CAZy annotation were statistically analyzed using the STAMP package^64^. Routinely, two-sided Fisher’s and/or ANOVA tests were used for hypothesis testing. Differences in proportions and 95% confidence intervals (CIs) were calculated according to the Newcombe-Wilson method. Multiple test corrections were done using the Storey FDR q-value or the Benjamini–Hochberg false discovery rate. A heat-map was constructed based on the RA by the Ward method and a dendrogram produced at a threshold of 0.9.

### Evaluation of gene clusters involved in (hemi)cellulose degradation

The metagenome assembly was performed using the CLC Genomics Workbench v4.0.3 with the following parameters: mismatch cost 2, insertion cost 3, deletion cost 3, length fraction 0.5 and similarity 0.9^65^,^66^. Each metagenome was assembled separately and contigs from 1W, 3W and 10W were used for subsequent assembly in order to obtain a dataset for RWS. The TWS consortia were analyzed similarly. In the RWS and TWS assembled datasets, genes were detected using the MetaGeneMark software^67^. Subsequently, these were annotated by Hidden Markov Models (HMM) based on CAZy family domains (v3) in the dbCAN platform using default parameters. Contigs ≥ 5 Kb with GH genes (specifically belonging to families GH2, GH3, GH29, GH31, GH43, GH92 and GH95) were retrieved and annotated using the RAST server^68^. The predicted GH were manually annotated using PSI-BLASTP against nr-NCBI database. Finally, to select contigs with GH “hotspots” and analyze their genomic contexts, we selected those contigs that had the followed parameters: 1) size ≥ 10 Kb, containing at least two GH genes, 2) size ≥ 35 Kb with at least one GH. G + C contents^69^, tetranucleotide frequencies (TTNF) and average nucleotide identities (ANIb) against different genomes^70^,^71^ were calculated in each selected contig.

## Additional Information

**How to cite this article**: Jiménez, D. J. *et al.* Unveiling the metabolic potential of two soil-derived microbial consortia selected on wheat straw. *Sci. Rep.* **5**, 13845; doi: 10.1038/srep13845 (2015).

## Supplementary Material

Supplementary figures

Supplementary Information

Supplementary Dataset 1

Supplementary Dataset 2

Supplementary Dataset 3

Supplementary Dataset 4

## Figures and Tables

**Table 1:** The most abundant and enriched CAZy families (top-15) in the RWS and TWS metagenomes (compared with FS1).

CAZy family	Metagenomes														Most common activities (EC Number)
	FS1		1W		3W		10W		1T		3T		10T		
	Log	RA	Log	RA	Log	RA	Log	RA	Log	RA	Log	RA	Log	RA	
PL17	0	0,017	1,135	0,236	1,071	0,203	1,122^c^	0,229	1,267	0,320	1,304	0,348	1,234^c^	0,296	Alginate lyase (EC 4.2.2.3); Oligoalginate lyase (EC 4.2.2.-)
CBM50	0	0,742	0,342	1,633	0,293	1,458	0,427^a^	1,983	0,548	2,622	0,426	1,980	0,228^b^	1,254	Carbohydrate-Binding Module (peptidoglycan and chitin binding module)
GT8	0	0,009	0,826	0,058	0,969	0,080	1,367^a^	0,201	0,980	0,082	0,263	0,016	0,757^b^	0,049	Lipopolysaccharide galacto(gluco)syltransferase; Homogalacturonan galacturonosyltransferase; Xylan glucuronyltransferase (EC 2.4.1.-)
GH117	0	0,009	1,593	0,338	1,513	0,281	1,528^c^	0,291	0,883	0,066	1,564	0,316	1,798^a^	0,542	Neoagarooligosaccharide hydrolase (EC 3.2.1.-)
GH92	0	0,311	1,060	3,564	0,978	2,955	0,861^b^	2,254	0,465	0,906	0,980	2,967	1,197^a^	4,895	Alpha-mannosidase (EC 3.2.1.24)
GH50	0	0,009	1,159	0,125	0,694	0,043	0,808^b^	0,055	1,306	0,175	1,144	0,120	1,264^b^	0,158	Beta-agarase (EC 3.2.1.81)
GH95	0	0,423	0,663	1,944	0,606	1,707	0,649^c^	1,886	0,181	0,642	0,636	1,831	0,797^a^	2,648	Alpha-1,2-L-fucosidase (EC 3.2.1.63); Alpha-L-fucosidase (EC 3.2.1.51)
GH20	0	0,570	0,523	1,900	0,462	1,652	0,384^b^	1,380	0,078	0,682	0,405	1,449	0,617^a^	2,359	Beta-hexosaminidase (EC 3.2.1.52); Lacto-N-biosidase (EC 3.2.1.140)
GH31	0	0,768	0,376	1,824	0,424	2,038	0,444^a^	2,136	0,446	2,148	0,502	2,439	0,492^c^	2,384	Alpha-glucosidase (EC 3.2.1.20); Alpha-xylosidase (EC 3.2.1.177); Alpha-mannosidase (EC 3.2.1.24)
GH2	0	1,839	0,336	3,987	0,410	4,722	0,417^a^	4,806	0,245	3,235	0,334	3,963	0,409^a^	4,715	*Beta-galactosidase (EC 3.2.1.23); *Beta-mannosidase (EC 3.2.1.25); Beta-glucuronidase (EC 3.2.1.31); Alpha-L-arabinofuranosidase (EC 3.2.1.55)
GH43	0	1,364	0,378	3,257	0,411	3,517	0,362^c^	3,141	0,210	2,214	0,366	3,166	0,348^a^	3,039	*Beta-xylosidase (EC 3.2.1.37); Alpha-L-arabinofuranosidase (EC 3.2.1.55); Xylanase (EC 3.2.1.8); Galactan 1,3-beta-galactosidase (EC 3.2.1.145)
GH1	0	0,656	0,260	1,192	0,424	1,740	0,457^a^	1,879	0,665	3,034	0,552	2,341	0,320^b^	1,370	*Beta-glucosidase (EC 3.2.1.21); Beta-galactosidase (EC 3.2.1.23); Beta-mannosidase (EC 3.2.1.25); Beta-glucuronidase (EC 3.2.1.31); Beta-xylosidase (EC 3.2.1.37); Beta-D-fucosidase (EC 3.2.1.38)
GH29	0	0,716	0,326	1,517	0,349	1,602	0,342^c^	1,574	−0,112	0,553	0,331	1,534	0,536^a^	2,461	Alpha-L-fucosidase (EC 3.2.1.51); Alpha-1,3/1,4-L-fucosidase (EC 3.2.1.111)
GH3	0	2,762	0,177	4,156	0,160	3,996	0,158^c^	3,974	0,139	3,804	0,083	3,343	0,035^b^	2,993	Beta-glucosidase (EC 3.2.1.21); Xylan 1,4-beta-xylosidase (EC 3.2.1.37); Beta-glucosylceramidase (EC 3.2.1.45); Beta-N-acetylhexosaminidase (EC 3.2.1.52); Alpha-L-arabinofuranosidase (EC 3.2.1.55)
GH13	0	7,242	−0,245	4,120	−0,258	4,001	−0,257^c^	4,008	−0,090	5,893	−0,099	5,760	−0,264^b^	3,940	Alpha-amylase (EC 3.2.1.1); Pullulanase (EC 3.2.1.41)

**Log**: Log10 X-fold increase in relative abundance.

**RA**: Relative abundance.

Negative values are subfamilies deselected comparatively with the FS1.

*Detected enzymatic activity in the secretome of both consortia at transfer-10 (Jimenez *et al.*, 2014b).

^a^Enriched along the transfers (selecting).

^b^Depleted along the transfers (deselecting).

^c^Neutral enrichment along the transfers.

**Table 2:** The most abundant/enriched CAZy family gene encoding metagenome fragments at transfer-10.

Consortia	CAZy family	Contig/gene ID	Length (bp)	NRC (%)^a^	BLASTx best hit [Taxa] (Accession number)	% QC	E	% I
10W	CBM50	Contig 1	1357	21 (7.3)	M23/M37 family cell wall endopeptidase [*Kluyvera ascorbata*] (KFD08719.1)	85	0.0	97
	GH2	Contig 5	2755	33 (4.7)	Beta-galactosidase [*Parabacteroides merdae*] (WP_005651198.1)	98	0.0	60
	GH20	Contig 3	1373	15 (7.5)	Beta-N-acetylhexosaminidase [*Sphingobacterium* sp.] (WP_031288206.1)	99	0.0	98
	GH95	Contig 5	1707	25 (9.1)	Fuc19 [*Sphingobacterium* sp.] (ACX30659.1)	99	0.0	92
	GH31	Contig 2	1572	25 (8.1)	Alpha-glucosidase [*Kluyvera ascorbata*] (KFD07596.1)	95	0.0	91
	GH3	Contig 1	2127	36 (6.2)	Periplasmic beta-glucosidase [*Kluyvera ascorbata*] (KFC99801.1)	96	0.0	95
	GH43	Contig 6	2144	32 (7.0)	Glycoside hydrolase family 43 [*Sphingobacterium* sp.] (CDT33327.1)	89	0.0	87
	GH1	Contig 4	1172	23 (8.4)	Beta-glucosidase/6-phospho-beta-glucosidase [*Kluyvera ascorbata*] (KFC94642.1)	93	0.0	94
	GH92	Contig 3	2533	29 (8.9)	Alpha-1,2-mannosidase [*Sphingobacterium* sp.] (CDT06451.1)	88	0.0	92
	GH29	Contig 1	1438	25 (11.0)	Alpha-L-fucosidase [*Sphingobacterium* sp.] (WP_031288978.1)	87	1E-144	99
10T	CBM50	Contig 1	1268	19 (5.3)	Peptidase [*Enterobacter aerogenes*] (WP_015705953.1)	85	0.0	95
	GH2	Contig 5	4302	228 (17.0)	Beta-galactosidase [*Sphingobacterium* sp.] (AIM35762.1)	92	0.0	78
	GH20	Contig 1	1753	88 (13.1)	Beta-N-acetylhexosaminidase [*Sphingobacterium* sp.] (WP_031287911.1)	93	0.0	98
	GH95	Contig 4	1792	84 (11.1)	Hypothetical protein [*Pedobacter borealis*] (WP_029274415.1)	99	0.0	77
	GH31	Contig 1	4119	189 (27.9)	Alpha-xylosidase [*Sphingobacterium* sp.] (AIM36311.1)	93	0.0	86
	GH3	Contig 4	2418	128 (15.0)	Beta-glucosidase [*Sphingobacterium* sp.] (CDS91967.1)	96	0.0	88
	GH43	Contig 4	4115	138 (15.9)	GH43D19 precursor [*Sphingobacterium* sp.] (ACX30655.1)	51	0.0	99
	GH1	Contig 3	1824	76 (19.5)	Beta-glucosidase A [*Sphingobacterium* sp.] (CDT05903.1)	73	0.0	83
	GH92	Contig 4	2597	131 (9.4)	Alpha-1,2-mannosidase [*Sphingobacterium* sp.] (CDT06451.1)	86	0.0	92
	GH29	Contig 7	2779	109 (15.5)	Alpha-1,3/4-fucosidase [*Sphingobacterium* sp.] (AIM37951.1)	94	0.0	90

**NRC**: Number of reads within the contig/gene; ^a^percentage of reads based on the total number of reads retrieved in each subfamily.

**QC**: Query coverage

**E**: E-value

**I**: Amino acid identity

**Table 3:** Glycosyl hydrolases detected by annotation using the CAZy database in thirteen putative novel predicted Bacteroidetes (hemi)cellulose utilization loci.

Contig ID (Consortia)	CL (bp)	NRC (%)^a^	CRC	CAZy family	Length (aa)	PSI-BLASTp best hit [Taxa] (Accession number)	% QC	E	% I
278 (RWS)	64,242	4,084 (0.19)	19X	GH29	596	Alpha-L-fucosidase [*Sphingobacterium spiritivorum*] (WP_003008132.1)	98	0.0	65
				GH29	477	Alpha-L-fucosidase [*Sphingobacterium spiritivorum*] (WP_003008126.1)	100	0.0	78
				GH3	765	Beta-glucosidase [*Sphingobacterium* sp.] (YP_004318346.1)	96	0.0	65
				GH92	717	Alpha-1,2-mannosidase [*Sphingobacterium spiritivorum*] (WP_003008094.1)	99	0.0	73
582 (RWS)	59,220	3,501 (0.16)	17.5X	GH43	360	Beta-xylosidase [*Bacteroides fragilis*] (WP_005778595.1)	95	0.0	80
				GH92	1032	Hypothetical protein [*Parabacteroides* sp.] (WP_010801039.1)	100	0.0	70
373 (RWS)	57,434	3,534 (0.17)	17X	GH92	657	Alpha-1 2-mannosidase [*Sphingobacterium spiritivorum*] (WP_002997981.1)	98	0.0	83
				GH92	766	Alpha-1,2-mannosidase [*Sphingobacterium* sp.] (YP_004319748.1)	98	0.0	67
				GH92	763	Alpha-1,2-mannosidase [*Sphingobacterium* sp.] (YP_004317288.1)	98	0.0	71
26 (RWS)	41,283	2,396 (0.11)	20X	GH29	326	Alpha-L-fucosidase [*Sphingobacterium spiritivorum*] (WP_003003741.1)	88	8e-174	82
				GH3	350	Beta-glucosidase [*Sphingobacterium* sp.] (YP_004316656.1)	94	0.0	78
262 (RWS)	38,022	2,177 (0.10)	17X	GH43	320	Arabinan endo-1,5-alpha-L-arabinosidase [*Sphingobacterium* sp.] (WP_002997231.1)	93	6e-176	78
				GH43	165	Beta-xylosidase [*Echinicola vietnamensis*] (YP_007226458.1)	93	1e-54	58*
939 (RWS)	23,370	1,210 (0.05)	14X	GH2	833	Glycoside hydrolase [*Dysgonomonas gadei*] (WP_006801003.1)	99	0.0	62
				GH43	641	Glycoside hydrolase [*Flavobacterium johnsoniae*] (YP_001195445.1)	98	0.0	66
				GH43	328	Glycosyl hydrolase family 32 [*Paraprevotella clara*] (WP_008623026.1)	88	1e-149	69
242 (RWS)	21,746	1,305 (0.06)	16.5X	GH2	670	Beta-galactosidase [*Parabacteroides goldsteinii*] WP_007653379.1	99	0.0	59*
				GH29	543	Alpha-1,3/4-fucosidase [*Capnocytophaga canimorsus*] (YP_004740108.1)	98	0.0	63
				GH95	747	Hypothetical protein [*Sphingobacterium spiritivorum*] (WP_003005855.1)	99	0.0	70
				GH2	1068	Beta-galactosidase [*Sphingobacterium spiritivorum*] (WP_002995396.1)	85	0.0	50*
248 (RWS)	19,539	1,037 (0.05)	15X	GH43	323	Alpha-N-arabinofuranosidase [*Dysgonomonas mossii*] (WP_006843656.1)	100	7e-176	72
				GH43	363	Glycoside hydrolase family protein [*Pedobacter saltans*] (YP_004274934.1)	96	0.0	77
				GH43	602	Glycosyl hydrolase [*Pedobacter saltans*] (YP_004274947.1)	99	0.0	75
				GH95	716	Alpha-L-fucosidase [*Pedobacter saltans*] (YP_004274942.1)	100	0.0	68
519 (TWS)	107,716	10,720 (0.43)	30X	GH2	439	Beta-galactosidase [*Bacteroides fragilis*] (WP_005786683.1)	95	3e-119	44*
				GH31	705	Glycosyl hydrolase [*Sphingobacterium spiritivorum*] (WP_003001890.1)	99	0.0	79
4309 (TWS)	39,914	4,462 (0.17)	23X	GH2	442	Beta-galactosidase [*Sphingobacterium spiritivorum*] (WP_002997393.1)	98	0.0	81
				GH95	507	Hypothetical protein [*Sphingobacterium* sp.] (WP_021190421.1)	100	0.0	98
316 (TWS)	25,776	2,588 (0.10)	22.5X	GH2	704	Hypothetical protein [*Parabacteroides* sp.] (WP_010803531.1)	99	0.0	59*
				GH2	567	Beta-galactosidase [*Parabacteroides merdae*] (WP_005649701.1)	97	1e-97	33*
				GH2	752	Beta-galactosidase [*Parabacteroides merdae*] (WP_005649701.1)	99	0.0	59*
3786 (TWS)	24,838	2,275 (0.09)	20.5X	GH2	427	Beta-galactosidase/beta-glucuronidase [*Flavobacterium* sp.] (WP_007809792.1)	90	1e-155	56*
				GH29	382	Glycoside hydrolase family protein [*Niastella koreensis*] (YP_005006468.1)	95	6e-81	39*
				GH95	807	Alpha-L-fucosidase [*Paludibacter propionicigenes*] (YP_004041891.1)	96	0.0	54*
				GH95	682	Hypothetical protein [*Sphingobacterium spiritivorum*] (WP_002994973.1)	99	0.0	70
1110 (TWS)	24,779	2,150 (0.08)	26X	GH43	140	Glycoside hydrolase [*Sphingobacterium* sp.] (YP_004317870.1)	78	9e-42	77
				GH43	531	Hypothetical protein [*Sphingobacterium* sp.] (WP_021189555.1)	55	0.0	99
				GH95	269	Alpha-L-fucosidase [*Pedobacter saltans*] (YP_004274942.1)	95	1e-83	53*
				GH95	430	Alpha-L-fucosidase [*Pedobacter salta*ns] (YP_004274942.1)	98	0.0	72

**CL**: Contig length.

**NRC**: Number of reads within the contig; ^a^ percentage of reads based on the total number of reads in RWS and TWS samples.

**CRC**: Coverage of reads per contig.

**QC**: Query coverage.

**E**: E-value.

**I**: Amino acid identity; *less than 60%.

## References

[b1] Ragauskas A. J. *et al.* The path forward for biofuels and biomaterials. Science 311, 484–489 (2006). 10.1126/science.1114736 16439654

[b2] Sims R. E., Mabee W., Saddler J. N. & Taylor M. An overview of second generation biofuel technologies. Bioresour. Technol. 101, 1570–1580 (2010). 10.1016/j.biortech.2009.11.046 19963372

[b3] Chandel A. K. & Singh O. V. Weedy lignocellulosic feedstock and microbial metabolic engineering: advancing the generation of ‘Biofuel’. Appl. Microbiol. Biotechnol. 89, 1289–1303 (2011). 10.1007/s00253-010-3057-6 21181146

[b4] Shang L. *et al.* Changes of chemical and mechanical behavior of torrefied wheat straw. Biomass. BioEnerg. 40, 63–70 (2012).

[b5] Limayem A. & Ricke S. C. Lignocellulosic biomass for bioethanol production: current perspectives, potential issues and future prospects. Prog. Energ. Combust. 38, 449–467 (2012).

[b6] Tumuluru J. S., Sokhansanj S., Hess J. R., Wright C. T. & Boardman R. D. A review on biomass torrefaction process and product properties for energy applications. Ind. Biotechnol. 7, 384–401 (2011).

[b7] de Souza R.W. Microbial degradation of lignocellulosic biomass in Sustainable degradation of lignocellulosic biomass - techniques, applications and commercialization (eds Chandel A. & Da Silva S.). Ch 9, 208–209 (de Vries, R. *et al.* 2001). Available at: http://www.intechopen.com/books/sustainable-degradation-of-lignocellulosic-biomass-techniques-applications-and-commercialization/microbial-degradation-of-lignocellulosic-biomass (Accessed: 10th December 2014).

[b8] Gao D. *et al.* Hemicellulases and auxiliary enzymes for improved conversion of lignocellulosic biomass to monosaccharides. Biotechnol. Biofuels. 4, 5 (2011). 10.1186/1754-6834-4-5 PMC3056733 21342516

[b9] Goldbeck R. *et al.* Development of hemicellulolytic enzyme mixtures for plant biomass deconstruction on target biotechnological applications. Appl. Microbiol. Biotechnol. 98, 8513–8525 (2014). 10.1007/s00253-014-5946-6 25077777

[b10] Xing M. N., Zhang X. Z. & Huang H. Application of metagenomic techniques in mining enzymes from microbial communities for biofuel synthesis. Biotechnol. Adv. 30, 920–929 (2012). 10.1016/j.biotechadv.2012.01.021 22306331

[b11] Cheng J. R. & Zhu M. J. A novel co-culture strategy for lignocellulosic bioenergy production: a systematic review. Int. J. Mod. Biol. Med. 1, 166–193 (2012).

[b12] Deangelis K. M. *et al.* Strategies for enhancing the effectiveness of metagenomic-based enzyme discovery in lignocellulolytic microbial communities. Bioenerg. Res. 3, 146–158 (2010).

[b13] Xia Y., Ju F., Fang H. H. & Zhang T. Mining of novel thermo-stable cellulolytic genes from a thermophilic cellulose-degrading consortium by metagenomics. PLoS One. 8, e53779 (2013). 10.1371/journal.pone.0053779 PMC3544849 23341999

[b14] Mori T., Kamei I., Hirai H. & Kondo R. Identification of novel glycosyl hydrolases with cellulolytic activity against crystalline cellulose from metagenomic libraries constructed from bacterial enrichment cultures. Springerplus. 3, 365 (2014). 10.1186/2193-1801-3-365 PMC4112031 25077068

[b15] Cantarel B. L. *et al.* The carbohydrate-active enzymes database (CAZy): an expert resource for glycogenomics. Nucleic. Acids. Res. 37, 233–238 (2009). 10.1093/nar/gkn663 PMC2686590 18838391

[b16] Allgaier M. *et al.* Targeted discovery of glycoside hydrolases from a switchgrass-adapted compost community. PLoS One. 5, e8812 (2010). 10.1371/journal.pone.0008812 PMC2809096 20098679

[b17] Suen G. *et al.* An insect herbivore microbiome with high plant biomass-degrading capacity. PLoS Genet. 6, e1001129 (2010). 10.1371/journal.pgen.1001129 PMC2944797 20885794

[b18] Shi W. *et al.* Comparative genomic analysis of the microbiome of herbivorous insects reveals eco-environmental adaptations: biotechnology applications. PLoS Genet. 9, e1003131 (2013). 10.1371/journal.pgen.1003131 PMC3542064 23326236

[b19] van der Lelie D. *et al.* The metagenome of an anaerobic microbial community decomposing poplar wood chips. PLoS One. 7, e36740 (2012). 10.1371/journal.pone.0036740 PMC3357426 22629327

[b20] Patel D. D. *et al.* Microbial and carbohydrate active enzyme profile of buffalo rumen metagenome and their alteration in response to variation in the diet. Gene. 545, 88–94 (2014). 10.1016/j.gene.2014.05.003 24797613

[b21] Cardoso A. M. *et al.* Metagenomic analysis of the microbiota from the crop of an invasive snail reveals a rich reservoir of novel genes. PLoS One. 7, e48505 (2012). 10.1371/journal.pone.0048505 PMC3486852 23133637

[b22] Wongwilaiwalin S. *et al.* Comparative metagenomic analysis of microcosm structures and lignocellulolytic enzyme systems of symbiotic biomass-degrading consortia. Appl. Microbiol. Biotechnol. 97, 8941–8954 (2013). 10.1007/s00253-013-4699-y 23381385

[b23] Deangelis K. M. *et al.* Metagenomes of tropical soil-derived anaerobic switchgrass-adapted consortia with and without iron. Stand. Genomic. Sci. 7, 382–398 (2013). 10.4056/sigs.3377516 PMC3764933 24019987

[b24] Jiménez D. J., Korenblum E. & van Elsas J. D. Novel multispecies microbial consortia involved in lignocellulose and 5-hydroxymethylfurfural bioconversion. Appl. Microbiol. Biotechnol. 98, 2789–2803 (2014). 10.1007/s00253-013-5253-7 24113822

[b25] Jiménez D. J., Dini-Andreote F. & van Elsas J. D. Metataxonomic profiling and prediction of functional behaviour of wheat straw degrading microbial consortia. Biotechnol. Biofuels. 7, 92 (2014). 10.1186/1754-6834-7-92 PMC4064818 24955113

[b26] Christensen H., Olsen R. A. & Bakken L. R. Flow cytometric measurements of cell volumes and DNA contents during culture of indigenous soil bacteria. Microb. Ecol. 29, 49–62 (1995). 10.1007/BF00217422 24186638

[b27] Meyer F. *et al.* The metagenomics RAST server: a public resource for the automatic phylogenetic and functional analysis of metagenomes. BMC Bioinform. 9, 1–8 (2008). 10.1186/1471-2105-9-386 PMC2563014 18803844

[b28] Huson D. H., Mitra S., Ruscheweyh H. J., Weber N. & Schuster S. C. Integrative analysis of environmental sequences using MEGAN4. Genome. Res. 21, 1552–1560 (2011). 10.1101/gr.120618.111 PMC3166839 21690186

[b29] Teeling H. & Glöckner F. O. Current opportunities and challenges in microbial metagenome analysis -a bioinformatic perspective. Brief. Bioinform. 13, 728–742 (2012). 10.1093/bib/bbs039 PMC3504927 22966151

[b30] Andreote F. D. *et al.* The microbiome of Brazilian mangrove sediments as revealed by metagenomics. PLoS One. 6, e38600 (2012). 10.1371/journal.pone.0038600 PMC3380894 22737213

[b31] Delmont T. O. *et al.* Structure, fluctuation and magnitude of a natural grassland soil metagenome. ISME J. 9, 1677–1687 (2012). 10.1038/ismej.2011.197 PMC3498926 22297556

[b32] Jiménez D. J. *et al.* Structural and functional insights from the metagenome of an acidic hot spring microbial planktonic community in the Colombian Andes. PLoS One. 12, e52069 (2012). 10.1371/journal.pone.0052069 PMC3522619 23251687

[b33] Ni J., Yan Q. & Yu Y. How much metagenomic sequencing is enough to achieve a given goal? Sci. Rep. 3, 1968 (2013). 10.1038/srep01968 PMC3678137 23752679

[b34] Větrovský T. & Baldrian P. The variability of the 16S rRNA gene in bacterial genomes and its consequences for bacterial community analyses. PLoS One. 8, e57923 (2013). 10.1371/journal.pone.0057923 PMC3583900 23460914

[b35] Aylward F. O. *et al.* Metagenomic and metaproteomic insights into bacterial communities in leaf-cutter ant fungus gardens. ISME J. 6, 1688–1701 (2012). 10.1038/ismej.2012.10 PMC3498920 22378535

[b36] Rodriguez G. M. & Smith I. Identification of an ABC transporter required for iron acquisition and virulence in *Mycobacterium tuberculosis*. J. Bacteriol. 188, 424–430 (2006). 10.1128/JB.188.2.424-430.2006 PMC1347291 16385031

[b37] Xu C. *et al.* Structure and regulation of the cellulose degradome in *Clostridium cellulolyticum*. Biotechnol. Biofuels. 6, 73 (2013). 10.1186/1754-6834-6-73 PMC3656788 23657055

[b38] Adav S. S., Cheow E. S., Ravindran A., Dutta B. & Sze S. K. Label free quantitative proteomic analysis of secretome by *Thermobifida fusca* on different lignocellulosic biomass. J. Proteomics. 75, 3694–3706 (2012). 10.1016/j.jprot.2012.04.031 22575269

[b39] Takasuka T. E., Book A. J., Lewin G. R., Currie C. R. & Fox B. G. Aerobic deconstruction of cellulosic biomass by an insect-associated *Streptomyces*. Sci. Rep. 3, 1030 (2013). 10.1038/srep01030 PMC3538285 23301151

[b40] Deangelis K. M. *et al.* Evidence supporting dissimilatory and assimilatory lignin degradation in *Enterobacter lignolyticus* SCF1. Front. Microbiol. 4, 280 (2013). 10.3389/fmicb.2013.00280 PMC3777014 24065962

[b41] Scully E. D. *et al.* Metagenomic profiling reveals lignocellulose degrading system in a microbial community associated with a wood-feeding beetle. PLoS One. 8, e73827 (2013). 10.1371/journal.pone.0073827 PMC3762729 24023907

[b42] Hottes A. K. *et al.* Transcriptional profiling of *Caulobacter crescentus* during growth on complex and minimal media. J. Bacteriol. 186, 1448–1461 (2004). 10.1128/JB.186.5.1448-1461.2004 PMC344409 14973021

[b43] Blanvillain S. *et al.* Plant carbohydrate scavenging through tonB-dependent receptors: a feature shared by phytopathogenic and aquatic bacteria. PLoS One. 2, e224 (2007). 10.1371/journal.pone.0000224 PMC1790865 17311090

[b44] Fernández-Gómez B. *et al.* Ecology of marine Bacteroidetes: a comparative genomics approach. ISME J. 5, 1026–1037 (2013). 10.1038/ismej.2012.169 PMC3635232 23303374

[b45] Kabisch A. *et al.* Functional characterization of polysaccharide utilization loci in the marine Bacteroidetes *‘Gramella forsetii*’ KT0803. ISME J. 8, 1492–1502 (2014). 10.1038/ismej.2014.4 PMC4069401 24522261

[b46] Ravcheev D. A., Godzik A., Osterman A. L. & Rodionov D. A. Polysaccharides utilization in human gut bacterium *Bacteroides thetaiotaomicron*: comparative genomics reconstruction of metabolic and regulatory networks. BMC Genomics. 14, 873 (2013). 10.1186/1471-2164-14-873 PMC3878776 24330590

[b47] Sonnenburg E. D. *et al.* Specificity of polysaccharide use in intestinal Bacteroides species determines diet-induced microbiota alterations. Cell. 141, 1241–1252 (2010). 10.1016/j.cell.2010.05.005 PMC2900928 20603004

[b48] Martens E. C. *et al.* Recognition and degradation of plant cell wall polysaccharides by two human gut symbionts. PLoS Biol. 9, e1001221 (2011). 10.1371/journal.pbio.1001221 PMC3243724 22205877

[b49] Larsbrink J. *et al.* A discrete genetic locus confers xyloglucan metabolism in select human gut Bacteroidetes. Nature. 506, 498–502 (2014). 10.1038/nature12907 PMC4282169 24463512

[b50] Qin J. *et al.* A human gut microbial gene catalogue established by metagenomic sequencing. Nature. 464, 59–65 (2010). 10.1038/nature08821 PMC3779803 20203603

[b51] Mhuantong W., Charoensawan V., Kanokratana P., Tangphatsornruang S. & Champreda V. Comparative analysis of sugarcane bagasse metagenome reveals unique and conserved biomass-degrading enzymes among lignocellulolytic microbial communities. Biotechnol Biofuels. 8, 16 (2015). 10.1186/s13068-015-0200-8 PMC4337096 25709713

[b52] Xu Z., Malmer D., Langille M. G., Way S. F. & Knight R. Which is more important for classifying microbial communities: who’s there or what they can do? ISME J. 12, 2357–2359 (2014). 10.1038/ismej.2014.157 PMC4260698 25171332

[b53] Kim H. T. *et al.* Overexpression and molecular characterization of Aga50D from *Saccharophagus degradans* 2-40: an exo-type beta-agarase producing neoagarobiose. Appl. Microbiol. Biotechnol. 86, 227–234 (2010). 10.1007/s00253-009-2256-5 19802606

[b54] Chi W. J., Chang Y. K. & Hong S. K. Agar degradation by microorganisms and agar-degrading enzymes. Appl. Microbiol. Biotechnol. 94, 917–930 (2012). 10.1007/s00253-012-4023-2 22526785

[b55] Singh K. M. *et al.* High potential source for biomass degradation enzyme discovery and environmental aspects revealed through metagenomics of indian buffalo rumen. Biomed. Res. Int. 2014, 267189 (2014). 10.1155/2014/267189 PMC4124647 25136572

[b56] Dai X. *et al.* Metagenomic insights into the fibrolytic microbiome in yak rumen. PLoS One. 7, e40430 (2012). 10.1371/journal.pone.0040430 PMC3396655 22808161

[b57] Mohagheghi A., Evans K., Chou Y. C. & Zhang M. Cofermentation of glucose, xylose, and arabinose by genomic DNA-integrated xylose/arabinose fermenting strain of *Zymomonas mobilis* AX101. Appl. Biochem. Biotechnol. 98-100, 885–898 (2002). 12018310

[b58] Kricka W., Fitzpatrick J. & Bond U. Metabolic engineering of yeasts by heterologous enzyme production for degradation of cellulose and hemicellulose from biomass: a perspective. Front. Microbiol. 5, 174 (2014). 10.3389/fmicb.2014.00174 PMC4001029 24795706

[b59] Wang Q., Garrity G. M., Tiedje J. M. & Cole J. R. Naive Bayesian classifier for rapid assignment of rRNA sequences into the new bacterial taxonomy. Appl. Environ. Microbiol. 73, 5261–5267 (2007). 10.1128/AEM.00062-07 PMC1950982 17586664

[b60] Claesson M. J. *et al.* Comparative analysis of pyrosequencing and a phylogenetic microarray for exploring microbial community structures in the human distal intestine. PLoS One. 4, e6669 (2009). 10.1371/journal.pone.0006669 PMC2725325 19693277

[b61] Yin Y. *et al.* dbCAN: a web resource for automated carbohydrate-active enzyme annotation. Nucleic. Acids. Res. 40, 445–451 (2012). 10.1093/nar/gks479 PMC3394287 22645317

[b62] Lombard V., Golaconda-Ramulu H., Drula E., Coutinho P. M. & Henrissat B. The carbohydrate-active enzymes database (CAZy) in 2013. Nucleic. Acids. Res. 42, 490–495 (2014). 10.1093/nar/gkt1178 PMC3965031 24270786

[b63] Huang Y., Niu B., Gao Y., Fu L. & Li W. CD-HIT Suite: a web server for clustering and comparing biological sequences. Bioinformatics. 5, 680–682 (2010). 10.1093/bioinformatics/btq003 PMC2828112 20053844

[b64] Parks D. H. & Beiko R. G. Identifying biologically relevant differences between metagenomic communities. Bioinformatics. 26, 715–721 (2010). 10.1093/bioinformatics/btq041 20130030

[b65] Mason O. U. *et al.* Metagenome, metatranscriptome and single-cell sequencing reveal microbial response to deepwater horizon oil spill. ISME J. 6, 1715–1727 (2012). 10.1038/ismej.2012.59 PMC3498917 22717885

[b66] Taş N. *et al.* Impact of fire on active layer and permafrost microbial communities and metagenomes in an upland Alaskan boreal forest. ISME J. 8, 1904–1919 (2014). 10.1038/ismej.2014.36 PMC4139727 24722629

[b67] Zhu W., Lomsadze A. & Borodovsky M. Ab initio gene identification in metagenomic sequences. Nucleic. Acids. Res. 38, e132 (2010). 10.1093/nar/gkq275 PMC2896542 20403810

[b68] Aziz R. K. *et al.* The RAST Server: rapid annotations using subsystems technology. BMC Genomics. 9, 75 (2008). 10.1186/1471-2164-9-75 PMC2265698 18261238

[b69] Gao F. & Zhang C. T. GC-Profile: a web-based tool for visualizing and analyzing the variation of GC content in genomic sequences. Nucleic. Acids. Res. 34, 686–691 (2006). 10.1093/nar/gkl040 PMC1538862 16845098

[b70] Teeling H., Meyerdierks A., Bauer M., Amann R. & Glöckner F. O. Application of tetranucleotide frequencies for the assignment of genomic fragments. Environ. Microbiol. 6, 938–947 (2004). 10.1111/j.1462-2920.2004.00624.x 15305919

[b71] Richter M. & Rosselló-Móra R. Shifting the genomic gold standard for the prokaryotic species definition. Proc. Natl. Acad. Sci. USA. 106, 19126–19131 (2009). 10.1073/pnas.0906412106 PMC2776425 19855009

