# Supplementary material for: Unveiling the metabolic potential of two soil-derived microbial consortia selected on wheat straw
Source: Sci Rep. 2015 Sep 7;5:13845. doi: 10.1038/srep13845 (PMC4561380; doi:10.1038/srep13845)
Supplement: Supplementary figures [file srep13845-s1.pdf]

Unveiling the metabolic potential of two soil-derived microbial consortia selected on wheat straw

Diego Javier Jiménez [1*], Diego Chaves-Moreno [2] and Jan Dirk van Elsas [1]

Affiliations

[1] Department of Microbial Ecology, Groningen Institute for Evolutionary Life Sciences, University of Groningen. Nijenborgh 7, 9747AG. Groningen, The Netherlands.

[2] Helmholtz–Centre for Infection Research, Inhoffenstr 7, 38124. Braunschweig, Germany.

*** Corresponding author:** Diego Javier Jiménez

Phone: +31 503632191; Fax: +31 503632348; Email: d.j.jimenez.avella@rug.nl

a)

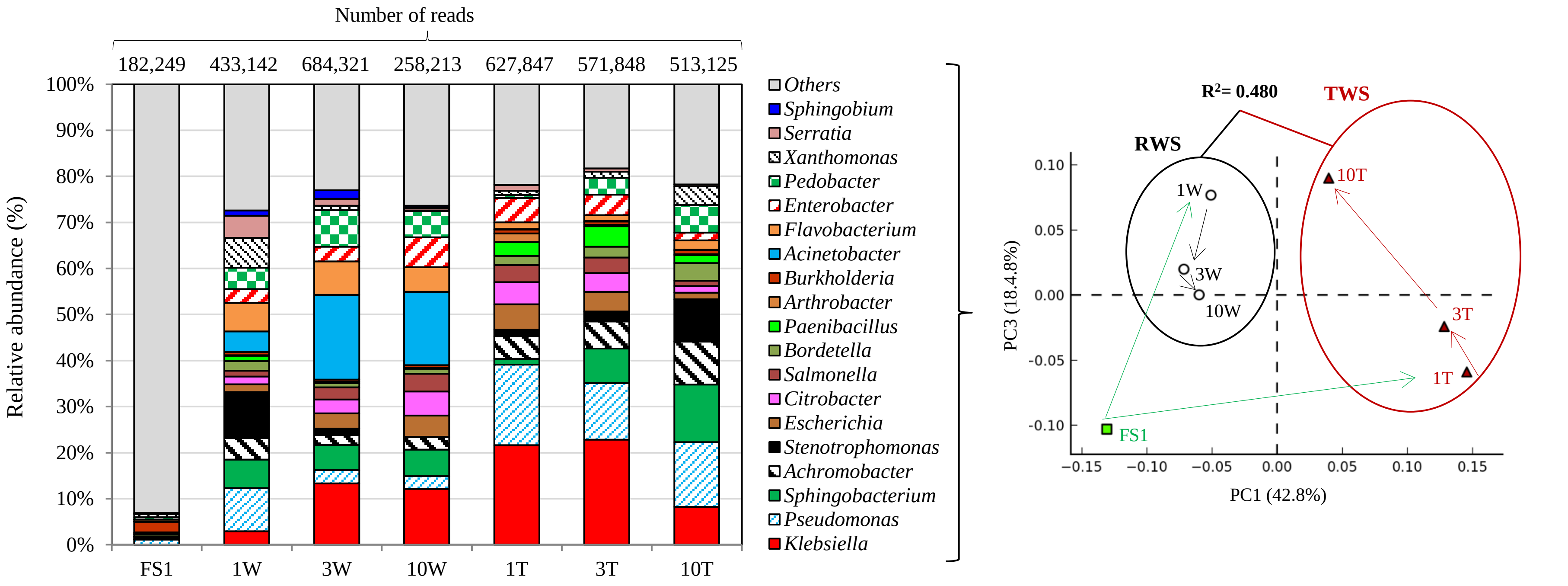

b)

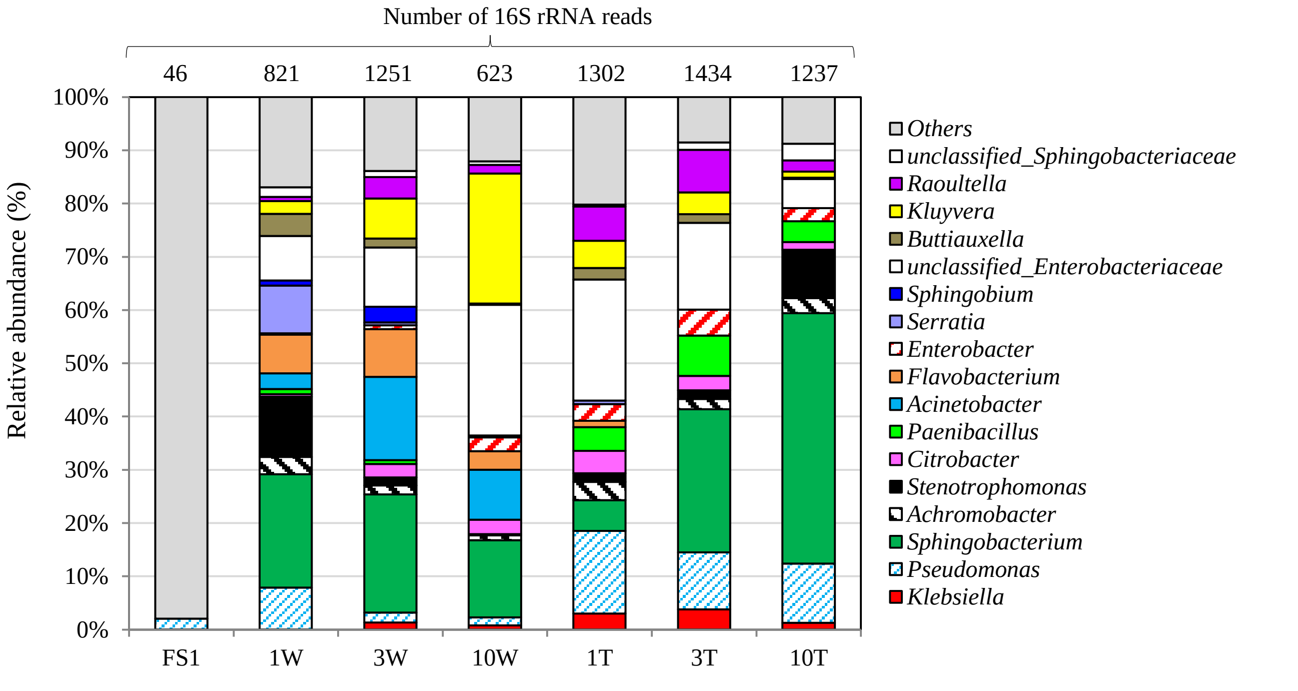

Supplementary Fig S1. Taxonomic profile of soil inoculum (FS1) and each metagenome that come from the three sequential transfers in RWS and TWS. **a)** Left: Taxonomic affiliation, based on protein-encoding reads (RefSeq database), of the most abundant bacterial genera in RWS and TWS samples, Right: PCA of the seven metagenomes using the classification at bacteria genus level; **b)** 16S rRNA reads classification using Classifier software

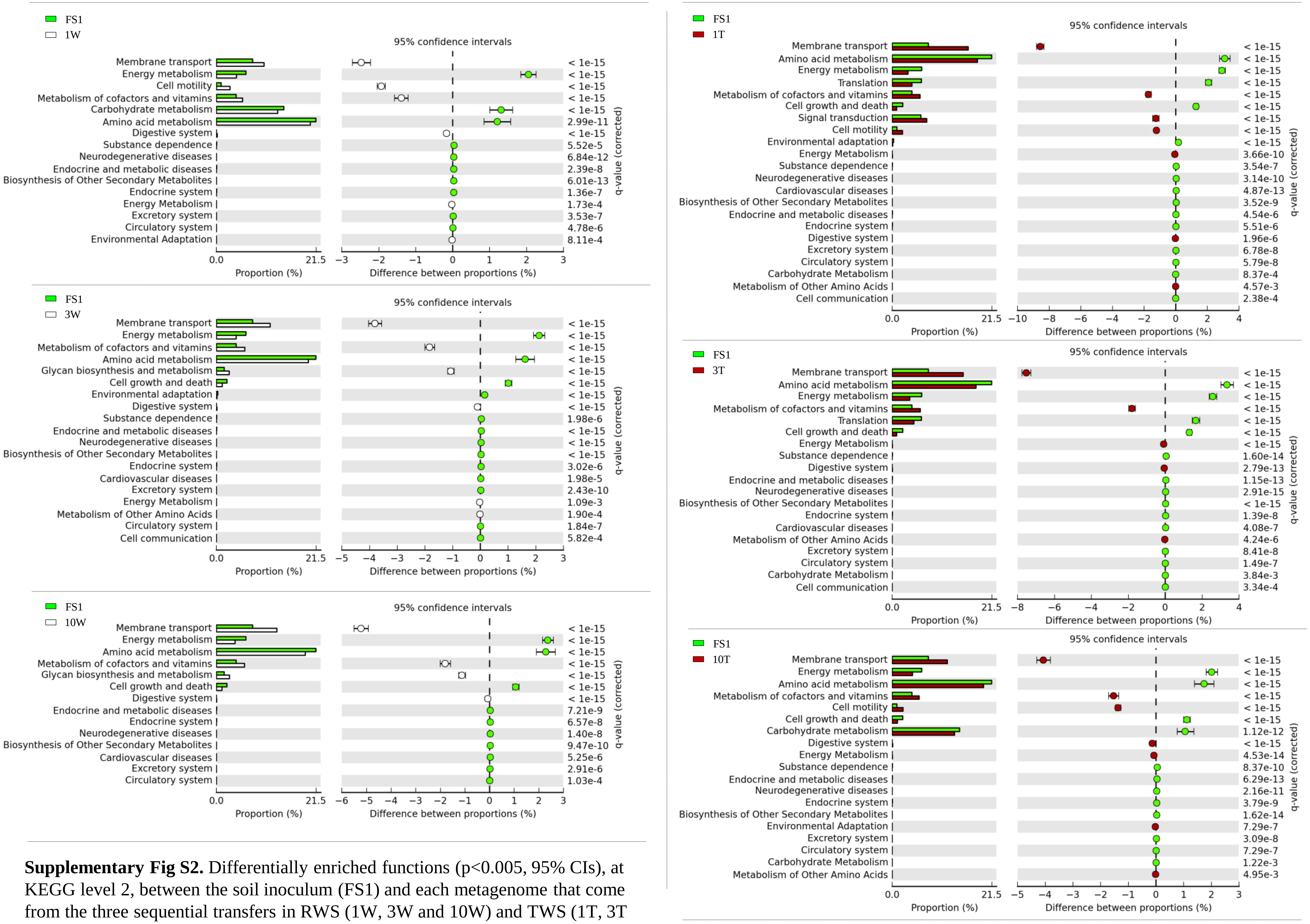

Supplementary Fig S2. Differentially enriched functions (p<0.005, 95% CIs), at KEGG level 2, between the soil inoculum (FS1) and each metagenome that come from the three sequential transfers in RWS (1W, 3W and 10W) and TWS (1T, 3T and 10T).

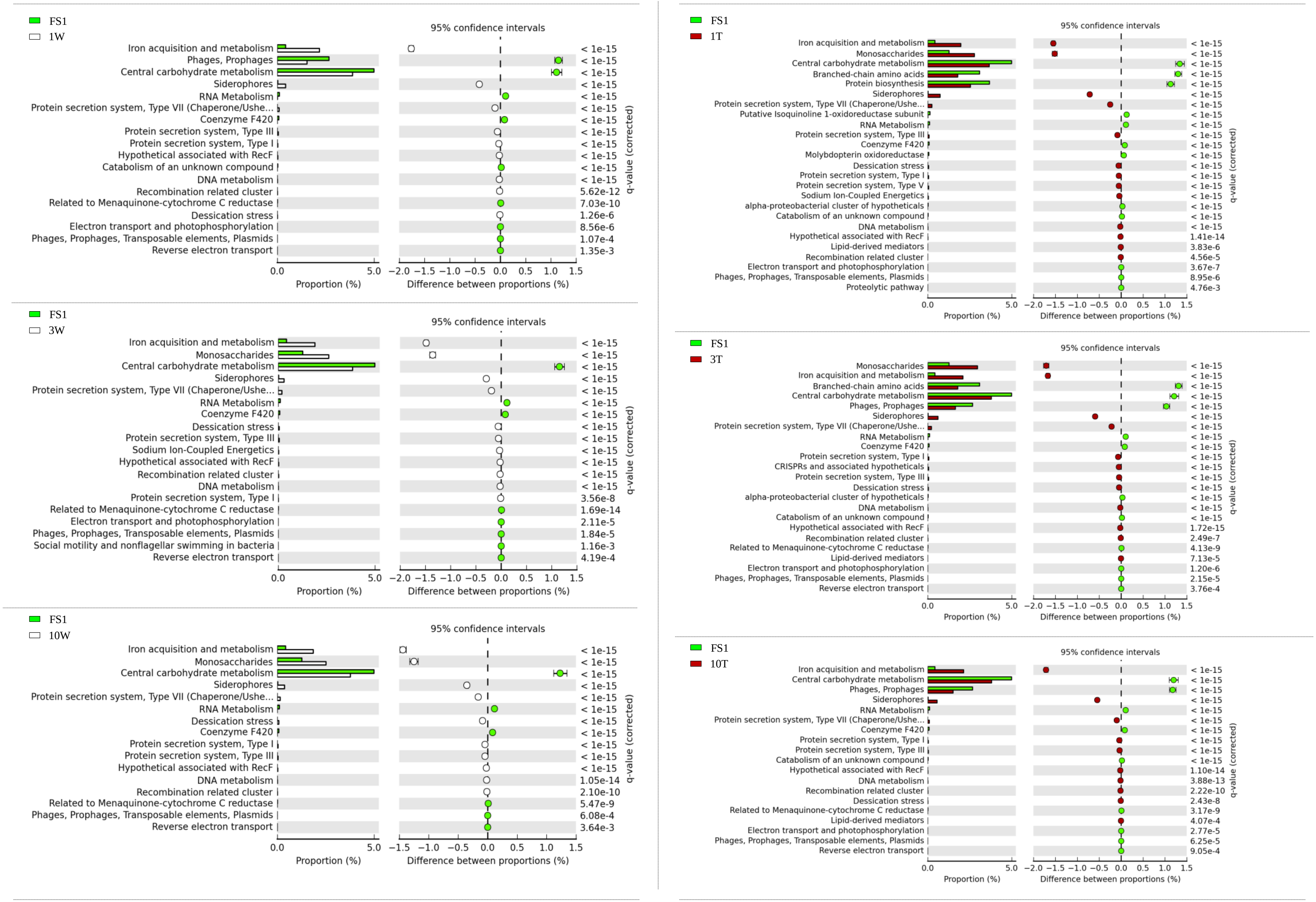

Supplementary Fig S3. Differentially enriched functions (p<0.005, 95% CIs), at SEED level 2, between the soil inoculum (FS1) and each metagenome that come from the three sequential transfers in RWS (1W, 3W and 10W) and TWS (1T, 3T and 10T).

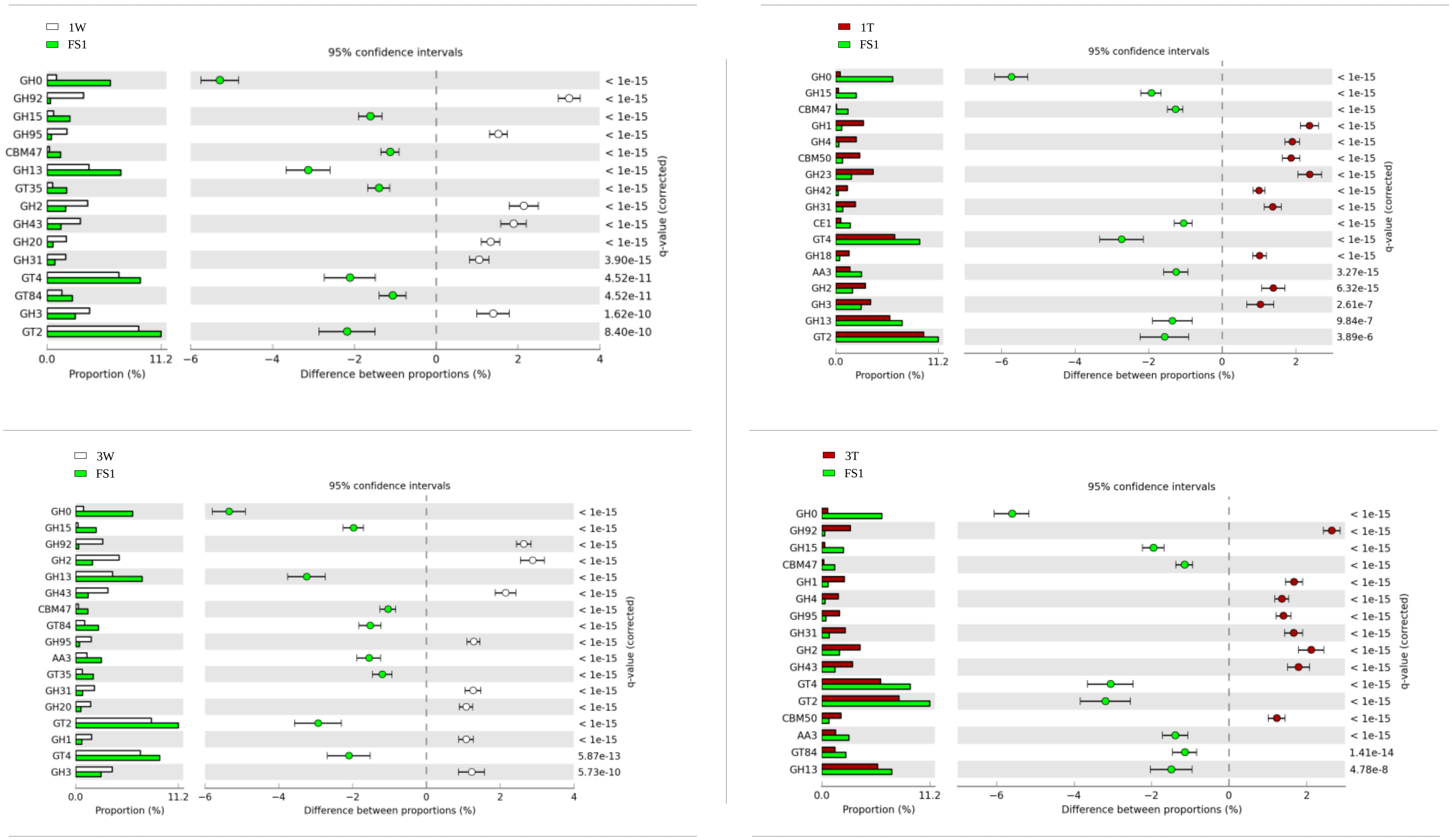

Supplementary Fig S4. Differentially enriched CAZy families ($p<0.005$, 95% CIs) between FS1 and 1W, 3W, 1T and 3T metagenomes.
