# Supplementary material for: Unveiling the metabolic potential of two soil-derived microbial consortia selected on wheat straw
Source: Sci Rep. 2015 Sep 7;5:13845. doi: 10.1038/srep13845 (PMC4561380; doi:10.1038/srep13845)
Supplement: Supplementary Information [file srep13845-s2.pdf]

Unveiling the metabolic potential of two soil-derived microbial consortia selected on wheat straw

Diego Javier Jiménez [1*], Diego Chaves-Moreno [2] and Jan Dirk van Elsas [1]

Affiliations

[1] Department of Microbial Ecology, Groningen Institute for Evolutionary Life Sciences, University of Groningen. Nijenborgh 7, 9747AG. Groningen, The Netherlands.

[2] Helmholtz–Centre for Infection Research, Inhoffenstr 7, 38124. Braunschweig, Germany.

*** Corresponding author:** Diego Javier Jiménez

Phone: +31 503632191; Fax: +31 503632348; Email: d.j.jimenez.avella@rug.nl

Supplementary figure legends

Supplementary Fig S1. Taxonomic profile of soil inoculum (FS1) and each metagenome that come from the three sequential transfers in RWS and TWS. **a)** Left: Taxonomic affiliation, based on protein-encoding reads (RefSeq database), of the most abundant bacterial genera in RWS and TWS samples, Right: PCA of the seven metagenomes using the classification at bacteria genus level; **b)** 16S rRNA reads classification using Classifier software.

Supplementary Fig S2. Differentially enriched functions ($p<0.005$, 95% CIs), at KEGG level 2, between the soil inoculum (FS1) and each metagenome that come from the three sequential transfers in RWS (1W, 3W and 10W) and TWS (1T, 3T and 10T).

Supplementary Fig S3. Differentially enriched functions ($p<0.005$, 95% CIs), at SEED level 2, between the soil inoculum (FS1) and each metagenome that come from the three sequential transfers in RWS (1W, 3W and 10W) and TWS (1T, 3T and 10T).

Supplementary Fig S4. Differentially enriched CAZy families ($p<0.005$, 95% CIs) between FS1 and 1W, 3W, 1T and 3T metagenomes.

Supplementary table legends

Table S1. Compositional profile (count matches, relative abundance and log_{10} X-fold increases) of genes involved in plant biomass deconstruction in FS1, 1W, 3W, 10W, 1T ,3T, and 10T metagenomes.

Table S2. Clustering (at 97% of nucleotide identity) and assembly of the metagenomic reads extracted from the most enriched CAZy families at transfer-10 and comparison with the FS1 metagenome.

Table S3. Glycosyl hydrolases detected in 145 contigs (≥ 5 Kb). The genes were detected using MetaGeneMark software, and the CAZy family domains were identify by Hidden Markov Models (HMM). The genes were manually annotated and taxonomically affiliated (based on the best hit) using PSI-BLASTP against nr-NCBI database.

Table S3. The tetranucleotide frequencies (TTNF) and the average nucleotide identities (AINb) calculated in thirteen selected contigs against 101 different genomes.
